# Determinants of catastrophic costs among households affected by multi-drug resistant tuberculosis in Ho Chi Minh City, Viet Nam: a prospective cohort study

**DOI:** 10.1186/s12889-023-17078-5

**Published:** 2023-12-03

**Authors:** Thi Anh Mai Pham, Rachel Forse, Andrew J. Codlin, Thi Hoang Yen Phan, Thanh Thi Nguyen, Nga Nguyen, Luan Nguyen Quang Vo, Phan Thuong Dat, Ha Dang Thi Minh, Lan Huu Nguyen, Hoa Binh Nguyen, Nhung Viet Nguyen, Miranda Bodfish, Knut Lönnroth, Tom Wingfield, Kristi Sidney Annerstedt

**Affiliations:** 1 WHO Collaborating Centre for Social Medicine and Tuberculosis, Department of Global Public Health Sciences, Karolinska Institute, Stockholm, Sweden; 2 Friends for International TB Relief, 1/21 Le Van Luong, Nhan Chinh, Thanh Xuan, Ha Noi, Viet Nam; 3 Centre for Development of Community Health Initiatives, 1/21 Le Van Luong, Nhan Chinh, Thanh Xuan, Ha Noi, Viet Nam; 4 Pham Ngoc Thach Hospital, 120 Hong Bang, Ward12, District 5, Ho Chi Minh City, Viet Nam; 5 National Lung Hospital/National TB Control Programme, 463 Hoang Hoa Tham, Vinh Phu, Ba Dinh, Ha Noi, Viet Nam; 6 University of Medicine and Pharmacy, Vietnam National University, Ha Noi, Viet Nam; 7 CDC Foundation, 600 Peachtree Street NE, Suite 1000, Atlanta, USA; 8 Clinical Sciences and International Public Health, Liverpool School of Tropical Medicine, Liverpool, L3 5QA Merseyside UK; 9 Tropical and Infectious Disease Unit, Liverpool University Hospital NHS Foundation Trust, Liverpool, L7 8XP Merseyside UK

**Keywords:** Multidrug-resistant tuberculosis, Catastrophic costs, Social protection, Patient Cost survey, Longitudinal design, Viet Nam

## Abstract

**Background:**

Globally, most people with multidrug-resistant tuberculosis (MDR-TB) and their households experience catastrophic costs of illness, diagnosis, and care. However, the factors associated with experiencing catastrophic costs are poorly understood. This study aimed to identify risk factors associated with catastrophic costs incurrence among MDR-TB-affected households in Ho Chi Minh City (HCMC), Viet Nam.

**Methods:**

Between October 2020 and April 2022, data were collected using a locally-adapted, longitudinal WHO TB Patient Cost Survey in ten districts of HCMC. Ninety-four people with MDR-TB being treated with a nine-month TB regimen were surveyed at three time points: after two weeks of treatment initiation, completion of the intensive phase and the end of the treatment (approximately five and 10 months post-treatment initiation respectively). The catastrophic costs threshold was defined as total TB-related costs exceeding 20% of annual pre-TB household income. Logistic regression was used to identify variables associated with experiencing catastrophic costs. A sensitivity analysis examined the prevalence of catastrophic costs using alternative thresholds and cost estimation approaches.

**Results:**

Most participants (81/93 [87%]) experienced catastrophic costs despite the majority 86/93 (93%) receiving economic support through existing social protection schemes. Among participant households experiencing and not experiencing catastrophic costs, median household income was similar before MDR-TB treatment. However, by the end of MDR-TB treatment, median household income was lower (258 [IQR: 0–516] USD vs. 656 [IQR: 462–989] USD; *p* = 0.003), and median income loss was higher (2838 [IQR: 1548–5418] USD vs. 301 [IQR: 0–824] USD; *p* < 0.001) amongst the participant households who experienced catastrophic costs. Being the household’s primary income earner before MDR-TB treatment (aOR = 11.2 [95% CI: 1.6–80.5]), having a lower educational level (aOR = 22.3 [95% CI: 1.5–344.1]) and becoming unemployed at the beginning of MDR-TB treatment (aOR = 35.6 [95% CI: 2.7–470.3]) were associated with experiencing catastrophic costs.

**Conclusion:**

Despite good social protection coverage, most people with MDR-TB in HCMC experienced catastrophic costs. Incurrence of catastrophic costs was independently associated with being the household’s primary income earner or being unemployed. Revision and expansion of strategies to mitigate TB-related catastrophic costs, in particular avoiding unemployment and income loss, are urgently required.

**Supplementary Information:**

The online version contains supplementary material available at 10.1186/s12889-023-17078-5.

## Background

Multidrug-resistant tuberculosis (MDR-TB), a form of TB with resistance to at least isoniazid and rifampicin, remains a global public health threat and a main contributor to deaths due to antimicrobial resistance (AMR) [1, 2]. Treatment regimens for MDR-TB are longer, more toxic, and more expensive than for drug-susceptible tuberculosis (DS-TB) [3]. For these reasons, while MDR-TB treatment success rates improved from 50 to 59% between 2012 and 2019, they remain persistently low [3].

Globally, the proportion of people with MDR-TB experiencing catastrophic costs, defined by the World Health Organization (WHO) as TB-related costs exceeding a threshold of 20% of annual household income, was almost twice as high compared to people with DS-TB (87% vs. 45%), perpetuating a vicious cycle of poverty and disease [3–5]. One of the three key performance indicators of the WHO End TB Strategy is to eliminate catastrophic costs by 2030, which is also reflected in the Sustainable Development Goals (SDG 3.8 and SDG 1.3) and the global agenda to reduce health inequalities [3]. Increasing out-of-pocket costs and lost income can adversely impact an affected person’s treatment adherence and potentially promote transmission of MDR-TB. Thus, people at risk of experiencing catastrophic costs need to be identified early and sufficiently protected against adverse treatment and socio-economic outcomes.

During the early days of the COVID-19 pandemic, health services were severely disrupted and access to TB care and prevention impeded. This distortion of the health system has meant that, for the first time since 2005, the TB mortality rates started to increase [3]. On an individual level, the pandemic and its associated lockdown measures increased job, income and productivity losses and aggravated poverty, especially in urban settings [6]. People with TB and their households, especially in low- and middle-income countries such as Viet Nam, continuously encountered financial uncertainty, and incurrence of catastrophic costs while coping with the outbreak [7].

Viet Nam is amongst the 20 countries with the highest estimated burden of MDR-TB [8]. Efforts to lower the MDR-TB burden include the introduction of the Programmatic Management of Drug-resistant Tuberculosis (PMDT) within its National TB Control Programme (NTP) in 2009 after which treatment enrolment and outcome improved [9]. National guidelines for the treatment of MDR-TB was aligned to the WHO consolidated guidelines on drug-resistant tuberculosis which was updated in 2020 and started a movement towards a shorter all-oral treatment regimen of nine months compared to the conventional longer treatment regimen of up to 20 months [10]. In the Vietnamese context, people receiving the shorter treatment regimen had significantly lower average treatment costs compared to people receiving the longer treatment regimen [11].

In 2016, the first national TB Patient Cost Survey (PCS) utilizing a standardized process and tool provided by the WHO for catastrophic costs surveillance was conducted in Viet Nam. The PCS reported higher direct non-medical costs, and lost income among participants with MDR-TB (58/735) resulting in a higher catastrophic costs prevalence of 98% compared to 60% among participants with DS-TB (677/735) [12]. Among the limitations of the Viet Nam national TB PCS was the use of a cross-sectional survey design. As opposed to longitudinal PCS implementation at multiple time points throughout treatment, cross-sectional TB PCSs rely on an extrapolation method to estimate costs incurred over the entire treatment duration, which can reduce the accuracy of the estimates [12]. Although a substantial number of studies have explored the factors associated with catastrophic costs among people with TB in general [4, 13–17], determining clinical and socioeconomic factors associated with catastrophic costs among MDR-TB-affected households remain a research and knowledge gap limiting effective design and implementation of national social protection schemes. To reduce the knowledge gap, this study fielded a longitudinal prospective TB PCS study to evaluate the factors associated with catastrophic costs among people with MDR-TB and their households in HCMC, Viet Nam.

## Methods

### Study design and participants

This study was a longitudinal prospective cohort study. People with MDR-TB initiating treatment during the period from October 2020 to July 2021 were referred to the Friends for International TB Relief (FIT) staff for potential recruitment. Participants with bacteriologically confirmed MDR-TB aged 18 or above, who planned to reside in the study area for the duration of 12 months and who received the 9-month treatment regimen were included. MDR-TB was bacteriologically confirmed using either antibiograms, genotype MTBDR plus (Hain) test or XPert MTB/RIF Assay. The individual treatment regimen for study participants who received phenotypic drug susceptibility testing (pDST) could change according to the results from the antibiograms. Yet, pDST is not a routine practice in Viet Nam. The participants included in this study received the same standard regimen (Table 1). Participants were excluded if the they were taking a 20-month regimen, another household member was already enrolled in the study or if they declined to provide informed consent.

**Table 1:** Glossary of operational definitions for tuberculosis disease, treatment, and cost calculations

**TB Disease**
Active TB disease: Person infected with a *Mycobacterium tuberculosis* which have overcome barriers of the immune system and actively growing in number, causing the person to develop TB symptoms and disease Latent TB disease: Person infected with *Mycobacterium tuberculosis* which have not yet overcome barriers of the immune system so that the person is protected against the development of TB symptoms and disease DS-TB: Disease with *Mycobacterium tuberculosis* susceptible to all first-line anti-TB drugs DR-TB: Disease with *Mycobacterium tuberculosis* resistant against one or more antibiotics RR-TB: Disease with *Mycobacterium tuberculosis* bacteria resistant against Rifampicin MDR-TB: Disease with *Mycobacterium tuberculosis* bacteria resistant against at least both isoniazid and rifampicin as first-line anti-TB drugs XDR-TB: TB caused by *Mycobacterium tuberculosis* (*M. tuberculosis*) strains that fulfil the definition of MDR/RR-TB and which are also resistant to any fluoroquinolone and at least one additional Group A drug (Group A drugs are the most potent group of drugs in the ranking of second-line medicines for the treatment of drug-resistant forms of TB using longer treatment regimens and comprise levofloxacin, moxifloxacin, bedaquiline and linezolid)
**TB Treatment**
9-month regimen: Short-term treatment regimen for MDR-TB usually lasting 9 consecutive months from treatment initiation to completion. The standard treatment regimen for participants of this study contained Clofazimine, Ethambutol, Isoniazid, Kanamycin, Levofloxacin, Prothionamide, and Pyrazinamide 20-month regimen: Long-term treatment regimen for MDR-TB usually lasting 20 consecutive months from treatment initiation to completion Intensive phase: Time period from treatment initiation until the start of the continuation phase usually lasting 4 to 6 consecutive months for the 9-month regimen Continuation phase: Time period immediately following the intensive treatment phase to the end of treatment usually lasting 5 consecutive months for the 9-month regimen Adverse TB treatment outcome: Death, treatment failure, loss to follow-up Pre-treatment: Time period from self-reported onset of TB-related symptoms until the initiation of treatment During treatment: Time period from treatment initiation until the end of the continuation phase
**TB Cost Calculations**
Direct cost: Out-of-pocket payment as sum of direct medical and direct non-medical costs during TB treatment Direct medical costs: Costs incurred due to medical examinations, medicine, consultation fees, radiography, and medical test Direct non-medical costs: Costs incurred due to transportation, accommodation, food, nutritional supplement, and relocation Indirect costs: Loss of productivity that the affected person and their household experience because of TB-related healthcare visits and hospitalisation during the TB treatment. It can be estimated by two alternative approaches: the output approach and the human capital approach Output approach: Estimate indirect costs based on income loss by subtracting household income before treatment from household income during treatment Human capital approach: Estimate indirect costs based on time loss by multiplying total hours lost due to care-seeking during the entire TB episode by hourly wage Annual household income: Annualized sum of income from each person within the same household. It can be self-reported by the interviewed person or predicted based on the presence of certain household assets Catastrophic costs: The proportion of population experiencing total TB-related costs that exceeds 20% of total household annual income or expenditure

The study was reported according to the Strengthening the Reporting of Observational Studies in Epidemiology (STROBE) cohort checklist (Additional file 1, Table S1).

### Survey setting

Viet Nam is a lower middle income country with a total population size of 97 million in 2020 [18]. The multidimensional poverty rate for the whole country decreased by nearly half from 9.2% in 2016 to 4.8% in 2020 [19]. The survey took place in 10 purposely selected districts of Ho Chi Minh City (HCMC), the largest and most densely populated city in Viet Nam comprising an estimated 11% of the country’s total population [20]. The HCMC monthly average income per capita (286 USD) in 2020 was higher compared to the national average of 186 USD [21].

At the time of the study, to finance TB care, the National TB Control Program (NTP) in Viet Nam provided TB drugs and diagnostics, and coordinated TB-specific social protection mechanisms. The Global Fund to Fight AIDS, Tuberculosis and Malaria supported the NTP’s MDR-TB program by investing in case detection, diagnosis, provision of quality-assured drugs and treatment services for MDR-TB, including social support such as subsidizing travel-related costs and nutritional support [22, 23]. Additional donors, clinical trials and implementation research studies also provided new drugs and support for Viet Nam’s MDR-TB program [24, 25]. Over 90% of Viet Nam’s general population has social health insurance (SHI) coverage [26], which subsidizes a specified range of diagnostic tests, treatments and inpatient hospitalization [27]. For the majority of the population, allowable medical expenditure is subsidized with a 20% out-of-pocket co-payment. For the uninsured, the MDR-TB program and out-of-pocket payments finance TB care [12].

In Viet Nam, TB services are provided across the three levels of health service delivery. The primary level includes healthcare facilities at the communes and districts such as district TB units or commune health centres. On the secondary level, provincial healthcare facilities such as the Pham Ngoc Thach Hospital in HCMC are in charge of administering DS-TB and MDR-TB related treatments for southern provinces or cities. The tertiary level covers healthcare facilities under the central government [28–30]. Individuals with MDR-TB in HCMC typically begin care and are notified at the secondary level, and are down-referred to the primary level for the completion of their treatment. While each district in HCMC is able to provide MDR-TB treatment, provincial level hospitals have the capacity for more specialized care and are responsible for monitoring of people with MDR-TB under treatment in the province.

### Data collection and sample size

We employed a locally-adapted WHO TB Patient Cost Survey (PCS) adjusted to the Vietnamese country context for people receiving MDR-TB treatment in HCMC for data collection. As people with TB can experience changes in cost and income at different phases throughout their treatment and to minimize recall bias, the survey was adapted longitudinally with three follow-up interviews similar to a previous study performed in HCMC [31]. The survey was refined and piloted among individuals with DR-TB prior to the study period (Additional file 3 and 4). Sampling for this study was opportunistic and the sample size was deemed appropriate given the WHO report on national surveys of costs faced by tuberculosis patients and their households 2015–2021 according to which the sample size for this study was larger than the average sample size of people with DR-TB for all countries and specifically larger than the National PCS in Vietnam (*n* = 59) [32].

Data included in this study were collected between October 2020 and April 2022. During the study and data collection period, HCMC was affected by a partial lockdown in 2020 until the first half of 2021 and another complete lockdown in July until October 2021 due to COVID-19 [33].

**Fig. 1:**
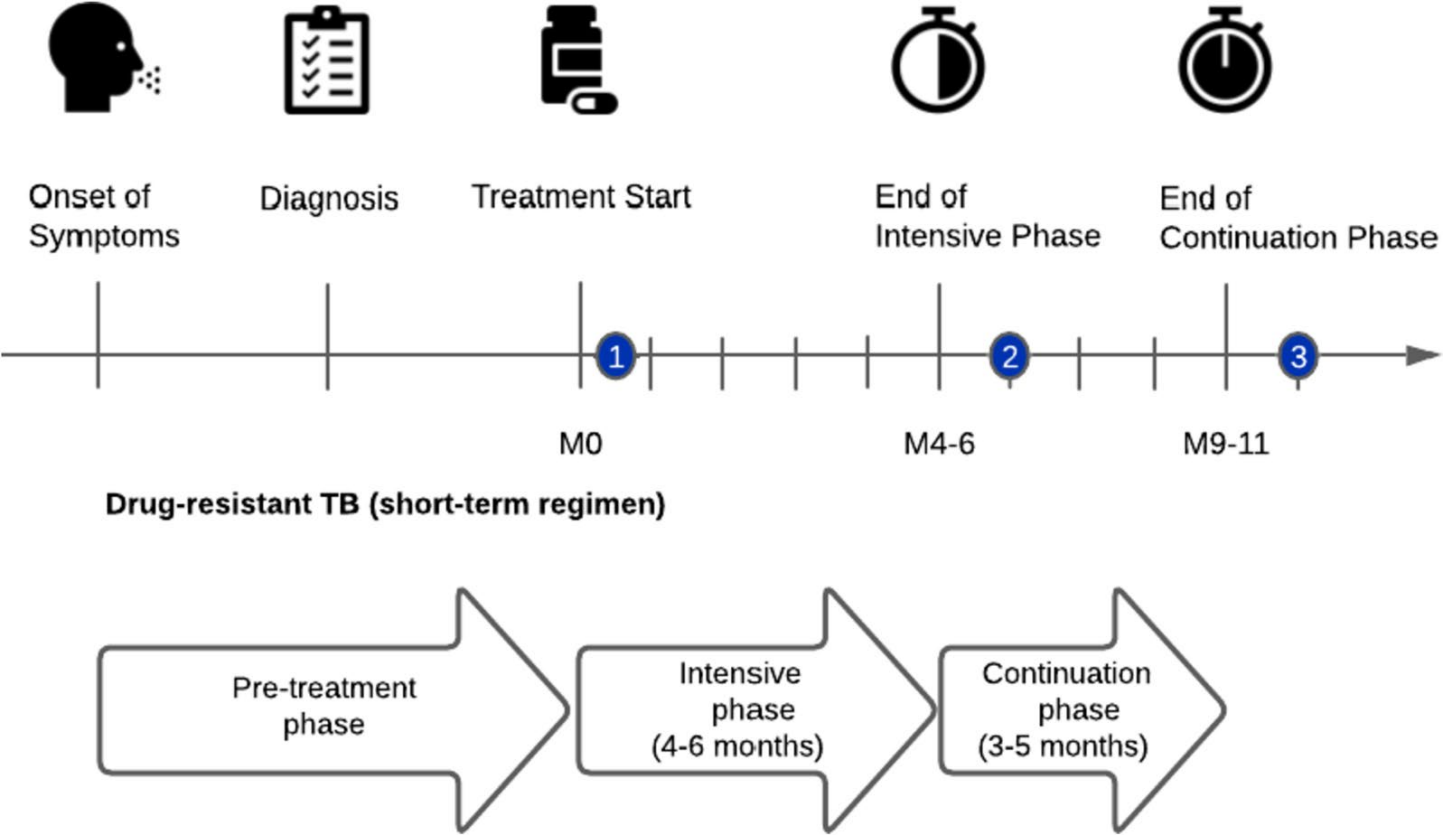
Timeline from symptom onset to treatment outcome for the 9-month treatment regimen of multidrug-resistant tuberculosis. Legend: Interview timepoints are highlighted in blue. Adapted from: Vo et al, 2021 [25]

Each participant was surveyed during three interview time points covering the entire treatment period from initiation to outcome assessment. The first interview was conducted in the intensive phase after at least 14 days and up to 6 weeks following treatment initiation and quantified pre-treatment costs from the onset of symptoms. The period included all costs incurred prior to diagnosis and up to that point in time. The second interview were completed at the end of the intensive phase (approximately the fifth month post-treatment initiation) and this included the costs between the first interview and second. The third interview took place during the continuation phase (approximately 10^th^ month post-treatment initiation) and this covered the period between the second and third interview., Information was collected on direct and indirect costs during the treatment for each phase (Fig. 1). The surveys were fielded by trained interview staff either in-person or via the phone at a place convenient for the interviewee such as the Pham Ngoc Thach Hospital, District TB Units, Commune Health Centres or at home. Responses were audio-recorded for subsequent quality control. If audio recordings were not available to fill in missing fields on the questionnaire paper during the interview, the quality of data was considered as insufficient and excluded from the final analysis. Data were then digitized using the ONA platform and the digitized data were compared against the paper survey to ensure data accuracy.

### Study variables

Potential exposure variables selected for this study were available through responses from the TB PCS and grouped into three categories: 1) Individual and household characteristics, i.e., general attributes of the person with MDR-TB and their household including sociodemographic, and clinical parameters; 2) MDR-TB treatment and care-seeking behaviour, i.e., the person’s treatment history and healthcare-seeking characteristics; and 3) Socioeconomic impact of MDR-TB treatment i.e., any changes in employment status, social consequences, impoverishment, coping strategies, and social protection mechanisms. In the first category, the wealth index was created as an indicator for the household’s socioeconomic status based on principle component analysis using all self-reported household assets and utilities available from the survey [34]. The wealth index was grouped into wealth terciles to facilitate interpretation.

As per WHO, catastrophic costs were defined as total TB related costs (direct medical, direct non-medical and indirect) exceeding 20% of the TB-affected household’s annual income [35] and presented as a binary variable (yes/no). The occurrence of catastrophic costs at the end of the treatment was estimated by the output approach for the primary analysis using total costs reported during the treatment [12, 35]. Other operational definitions of TB disease, treatment and cost calculations used in this study are described in Table 1. Missing values for single cost components were imputed with the median if less than 10% were missing. All equations used in the primary and sensitivity analysis by which catastrophic costs were calculated in this study are presented in Table 2. A description on how primary and sensitivity analyses were performed can be found in the appendix (Additional file 5).

**Table 2:** Collection of equations used for catastrophic costs calculation

**Primary analysis:** using the output approach including amount of vouchers and social welfare payments or cash transfers as income
$$catastrophic\;costs\;if\;\frac{\left(m-s\right)+n+i}{r+c+v}*100>20\%$$
**Alternative I:** using the output approach excluding amount of vouchers and social welfare payments or cash transfers
$$catasrophic\;costs\;if\;\frac{\left(m-s\right)+n+i}{r}*100>20\%$$
**Alternative II:** using the output approach with asset-based income instead of self-reported income
$$catastrophic\;costs\;if\;\frac{\left(m-s\right)+n+i}{a+c+v}*100>20\%$$
**Alternative III:** using the human capital approach
$$catastrophic\;costs\;if\;\frac{\left(m-s\right)+n+h}{r+c+v}*100>20\%$$
**Alternative IV:** using the direct cost only
$$catasrtophic\;costs\;if\;\frac{\left(m-s\right)+n}{r+c+v}*100>20\%$$
where: m = direct medical costs n = direct non-medical costs i = indirect cost as income loss h = indirect cost as product of working hours lost and hourly rate s = reimbursements from private health insurance v = amount from any vouchers received c = amount from any social welfare payments and cash transfers r = Annual self-reported household income a = Annual asset-based household income

For the breakdown of total costs during the entire treatment, periodically incurred cost components were multiplied by the frequency of drug-pickup or directly observed treatment (DOT) visits per month of treatment. Costs during the continuation phase contained all cost data collected during the second and third interview. Changes in costs between the two interview time points were assumed to occur after half of the continuation phase duration. Costs and income were converted from the local currency to USD (VND1 = USD 0.000043, 2020–2022, OANDA) [36].

### Data analysis

Descriptive analysis was performed including frequencies and proportions for categorical variables or mean and standard deviations (SD) for normally distributed continuous variables and median and interquartile ranges (IQR) for non-normally distributed continuous variables. Pearson’s Chi-squared test, Fisher’s exact test, t-test and Wilcoxon rank sum test were used as appropriate [37]. P-values below 0.05 were considered statistically significantly. Univariable logistic regression was applied to estimate the association’s magnitude, in form of crude odds ratio (OR) and their significance. The final multivariable logistic regression model was adjusted for age, gender, and education status as potential confounders based on an a priori conceptual framework [5, 38, 39] (Additional File 2, Figure S3). Hosmer–Lemeshow Goodness of fit was tested. The variance of inflation factors (VIF) was used to assess for multicollinearity with a threshold of 10. No multicollinearity was detected among variables included the multivariable regression analysis (mean VIF = 1.25). The sensitivity of catastrophic costs estimation was evaluated by additional analysis which included different calculation approaches (Table 2) and alternative catastrophic costs thresholds from 1 to 200% to define catastrophic costs. Data analysis was performed in Stata v17 (StataCorp, College Station, TX, USA).

### Ethical considerations

Ethical approvals were granted by the Pham Ngoc Thach Hospital Institutional Review Board (1225/PNT-HĐĐĐ) in September 2020. Informed consent was obtained from each participant. Only pseudonymised data were used for this analysis.

## Results

### Study participants

**Fig. 2:**
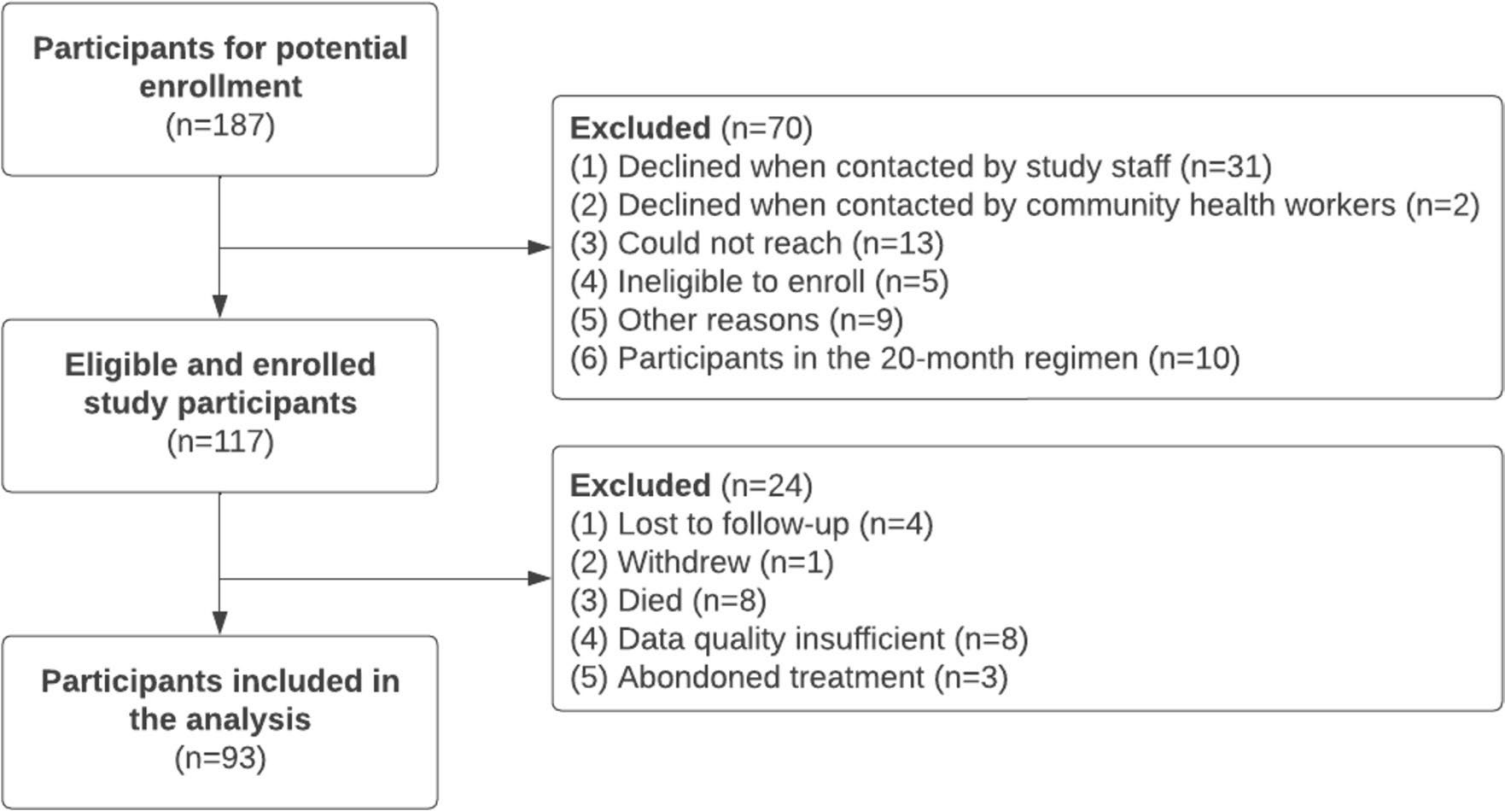
Flow chart from identification to inclusion of study participants

One hundred and eighty-seven individuals were eligible for recruitment in the survey and 117 individuals receiving a nine-month regimen were enrolled as participants, of whom 93/117 (79%) completed the final interview and were included in the data analysis (Fig. 2). The reasons people were not enrolled were declining to participate (*n* = 33), not contactable for the first interview (*n* = 13), incorrectly assigned as eligible according to inclusion and exclusion criteria (*n* = 5), and other reasons such as unreachable due to the COVID-19 lockdown (*n* = 9). Twenty-four participants who enrolled in the survey were excluded from this analysis because they were lost to follow-up (*n* = 7), withdrew from the study (*n* = 1), died (*n* = 8), or the quality of the data was insufficient (*n* = 8) (Fig. 2). Of the 24 participants excluded, data on baseline characteristics was available for 16 participants showing a significantly older age (54 [SD: 13] vs. 44 [SD: 15], *p* = 0.012) and lower wealth (Poorest Wealth Tercile: 69% vs. 31%) compared to participants who completed the final interview.

### Baseline characteristics and catastrophic costs prevalence

Among the included participants, 70/93 (75%) of the individuals were male and the average cohort age was 44 years (SD: 15) (Table 3). Participants had on average nine years (SD: 5) of education with 50% having completed secondary school or higher education. The majority of study participants were covered by social health insurance (SHI) (79/93 [85%]).

**Table 3:** Baseline characteristics of study participants receiving multidrug-resistant tuberculosis treatment by experiencing catastrophic costs

Characteristics	No catastrophic costs (*N* = 12)	Catastrophic	Total	*p*-value^*,**,***,****^
		**costs (** ***N*** ** = 81)**	**(** ***N*** ** = 93)**	
	n (%),	n (%),	n (%),	
	mean (SD) or	mean (SD) or	mean (SD) or	
	median (IQR)	median (IQR)	median (IQR)	
**Individual and household characteristics**				
Age				
Years of age, mean (SD)	44.3 (20.0)	43.5 (14.0)	43.6 (14.8)	0.851^***^
Gender				0.137^*^
Female	5 (41.7)	18 (22.2)	24 (25.5)	
Male	7 (58.3)	63 (77.8)	70 (75.3)	
Participant’s education level				**0.046** ^*^
No formal education	1 (8.3)	17 (21.0)	18 (19.4)	
Up to primary school only	1 (8.3)	28 (34.6)	29 (31.2)	
Completed secondary school and above	10 (83.3)	36 (44.4)	46 (49.5)	
Years of education, mean (SD)	11.8 (4.6)	8.1 (5.1)	8.6 (5.2)	**0.018** ***
Social health insurance (SHI)				
Covered by SHI	10 (83.3)	69 (85.2)	79 (84.9)	1.000^*^
Household status				
Head of household	4 (33.3)	36 (44.4)	40 (43.0)	0.468^**^
Primary income earner before disease	2 (16.7)	40 (49.4)	42 (45.2)	**0.034** **
Household size				
Number of household members, mean (SD)	5.0 (1.7)	4.1 (2.2)	4.3 (2.2)	0.2^***^
Number of rooms in the house, median (IQR)	3.0 (2.5–4.0)	3.0 (2.0–4.0)	3.0 (2.0–4.0)	0.459^****^
Number of people per room, median (IQR)	1.5 (1.0–2.3)	1.3 (1.0–1.8)	1.3 (1.0–1.75)	0.437^****^
Crowding^a^	6 (50.0)	36 (43.9)	42.0 (44.7)	0.692^**^
Household wealth^b^				**0.002** ^*^
Tercile 1 (least wealthy)	0 (0.0)	31 (38.3)	31 (33.3)	
Tercile 2	3 (25.0)	28 (34.6)	31 (33.3)	
Tercile 3 (wealthier)	9 (75.0)	22 (27.2)	31 (33.3)	
Wealth index, mean (SD)	1.6 (1.4)	-0.2 (2.0)	-7.8*10^–09^ (2.0)	**0.003** ***
**MDR-TB treatment and care seeking behaviours**				
Hospitalisation status				
Hospitalised due to TB before the treatment	4 (33.3)	32 (39.5)	36 (38.7)	0.761^*^
Hospitalised due to TB at any moment	2 (16.7)	17 (21.0)	19 (20.4)	1.000^*^
during the treatment				
History of TB				
Previous TB episodes	7 (58.3)	39 (48.2)	46 (49.5)	0.510^**^
TB treatment of other household members				
Other household members receiving TB treatment	0 (0.0)	2 (2.5)	2 (2.2)	1.000^*^
Comorbidities				
Presence of comorbidities	6 (50.0)	36 (44.4)	42 (45.2)	0.718^**^
HIV	0 (0.0)	8 (9.9)	8 (8.6)	0.590^*^
Nutritional supplements				
Use of nutritional supplements	12 (100.0)	79 (97.5)	91 (97.9)	1.000^*^
Treatment duration				
Treatment duration (in months), median (IQR)	9.0 (9.0–9.0)	9 (8.0–9.0)	9.0 (8.0–9.0)	0.428^****^
Number of visits for ambulatory care				
Pre-TB, median (IQR)	6.5 (5.5–11.5)	9.0 (6.0–20.0)	9.0 (5.0–18.0)	0.355^****^
Directly observed therapy, median (IQR)	103.9 (103.9–116.9)	103.9 (90.9–103.9)	103.9 (103.9–103.9)	0.506^****^
Drug pick-up, median (IQR)	22.8 (13.9–53.7)	21.3 (14.7–30.3)	21.7 (14.2–30.3)	0.904^****^
Follow-up, mean (SD)	5.8 (2.1)	6.3 (2.1)	6.2 (2.1)	0.400^***^
**Socioeconomic impact of MDR-TB treatment**				
Pre-treatment employment status				0.059^*^
Unemployed	2 (16.7)	10 (12.4)	12 (12.9)	
Formal Paid Work	2 (16.7)	15 (18.5)	17 (18.3)	
Informal Paid Work	2 (16.7)	40 (49.4)	42 (45.2)	
Other	6 (50.0)	16 (19.8)	22 (23.7)	
Employment status during treatment				
Change to unemployment at the beginning of treatment	1 (8.3)	44 (54.3)	45 (48.4)	**0.003** **
Social consequences				
Lost job due to MDR-TB illness	5 (41.7)	61 (75.3)	66 (71.0)	**0.035** ^*^
Food Insecurity	0 (0.0)	12 (14.8)	12 (12.9)	0.353^*^
Social exclusion or stigma	0 (0.0)	13 (16.1)	13 (14.0)	0.206^*^
Isolation from family, self-limiting contact or communication with others	11 (91.7)	67 (82.7)	78 (83.9)	0.683^*^
Impoverishment				
Poverty headcount before MDR-TB treatment	1 (8.3)	4 (4.9)	5 (5.4)	0.507^*^
Pushed below poverty line after MDR-TB treatment	2 (16.7)	24 (29.6)	26 (28.0)	0.500^*^
Coping strategies				
Any form of coping strategy	1 (8.3)	53 (65.4)	54 (58.1)	** < 0.001** **
Taking loans	1 (8.3)	48 (59.3)	49 (52.7)	**0.001** **
Selling assets	0 (0.0)	18 (22.2)	18 (19.4)	0.114^*^
Social protection				
Any form of social welfare payments or cash transfers received	8 (66.7)	78 (96.3)	86 (92.5)	**0.005** ^*^
Sick leave	1 (8.3)	10 (12.4)	11 (11.8)	1.000^*^
Cash transfer from governmental agencies or policies	1 (8.3)	17 (21.0)	18 (19.4)	0.450^*^
Cash transfer from other NGOs	8 (66.7)	76 (93.8)	84 (90.3)	**0.015** *
Any vouchers received	7 (58.3)	71 (87.7)	78 (83.9)	**0.022** ^*^
Vouchers from governmental agencies	7 (58.3)	62 (76.5)	69 (74.2)	0.286^*^
Vouchers from any NGO	0 (0.0)	3 (3.7)	3 (3.2)	1.000^*^
Voucher from private donation	0 (0.0)	20 (24.7)	20 (21.5)	0.063^*^
Voucher from other sources	0 (0.0)	3 (3.7)	3 (3.2)	1.000^*^

Estimation of catastrophic costs using the output approach at a 20% threshold

*Abbreviations*: *HIV* Human immunodeficiency virus, *TB* Tuberculosis, *MDR-TB* Multidrug-resistant TB, *SD* Standard deviation, *IQR* Interquartile range

^*^(1-sided) Fischer’s exact test for proportions (any cell with expected frequency < 5 in the contingency table), ^**^ Pearson’s Chi-squared test for proportions, ^***^ t test for means, ^****^ Wilcoxon rank sum test for median

^a^Crowding is defined as number of household members per room exceed the cohort median

^b^Wealth index and its categorization in wealth terciles generated using principal component analysis on 30 household assets and characteristics

By the end of MDR-TB treatment, 81/93 participants (87.1% [95%CI: 78.5–92.6]) experienced catastrophic costs at a 20% threshold. Compared to people with MDR-TB and their households who did not experience catastrophic costs, people with MDR-TB and their households experiencing catastrophic costs had fewer years of education (8 [SD: 5] vs. 12 [SD: 5] years; *p* = 0.017) and were more likely to be the primary income earners of the household before MDR-TB diagnosis (40/81 [49%] vs. 2/12 [17%]; *p* = 0.034) or to belong to the poorest tercile (31/81 [38%] vs. 0/12 [0%]; *p* = 0.002).

### Treatment, care-seeking behaviour, and socioeconomic impact

All participants who had another household member receiving TB treatment (2/93 [2%]) or who were co-infected with HIV (8/93 [10%]) experienced catastrophic costs. About half of the study population (46/93 [50%]) had a previous episode of TB.

People with MDR-TB who experienced catastrophic costs had higher rates of being unemployed after falling ill with TB (44/81 [54%] vs. 1/12 [8%]; *p* = 0.003) and losing their job as a consequence of MDR-TB illness at some point during the treatment (61/81 [75%] vs. 5/12 [42%]; *p* = 0.035). All participants who self-reported experiences of social exclusion or stigma due to TB illness (13/93 [16%]) also experienced catastrophic costs. People with MDR-TB who experienced catastrophic costs had higher rates of using coping strategies such as taking loans (48/81 [59%] vs. 1/ 12 [8%]; *p* = 0.001) than those who did not experience catastrophic costs. People from the poorest wealth tercile had three-fold higher rates of using coping strategies during their treatment than people from the highest wealth tercile (25/32 [78%] vs. 9/31 [29%]; *p* < 0.001). At the end of the treatment the number of people taking loans within the overall cohort was almost twice as high compared to the number of people taking loans at the beginning of the treatment (33/93 [35%] vs. 19/93 [20%]).

In terms of social protection mechanisms, people with MDR-TB who experienced catastrophic costs had higher rates of receiving cash transfers from non-governmental organizations (NGOs) (76/81 [94%] vs. 8/12 [67%]; *p* = 0.015), or any kind of vouchers (71/82 [88%] vs. 7/12 [58%]; *p* = 0.022). At the beginning of the treatment, only 14/81 [17%] of people experiencing catastrophic costs have received vouchers whereas towards the end of the treatment the majority was covered (63/81 [78%]) (Additional file 2, Fig. S1). Vouchers were given in form of travel support, food support, or one-time and infrequent vouchers, as well as in-kind support such as gifts from relatives and friends who are not members of the household, or charity.

### Income changes and cost consequences

**Fig. 3:**
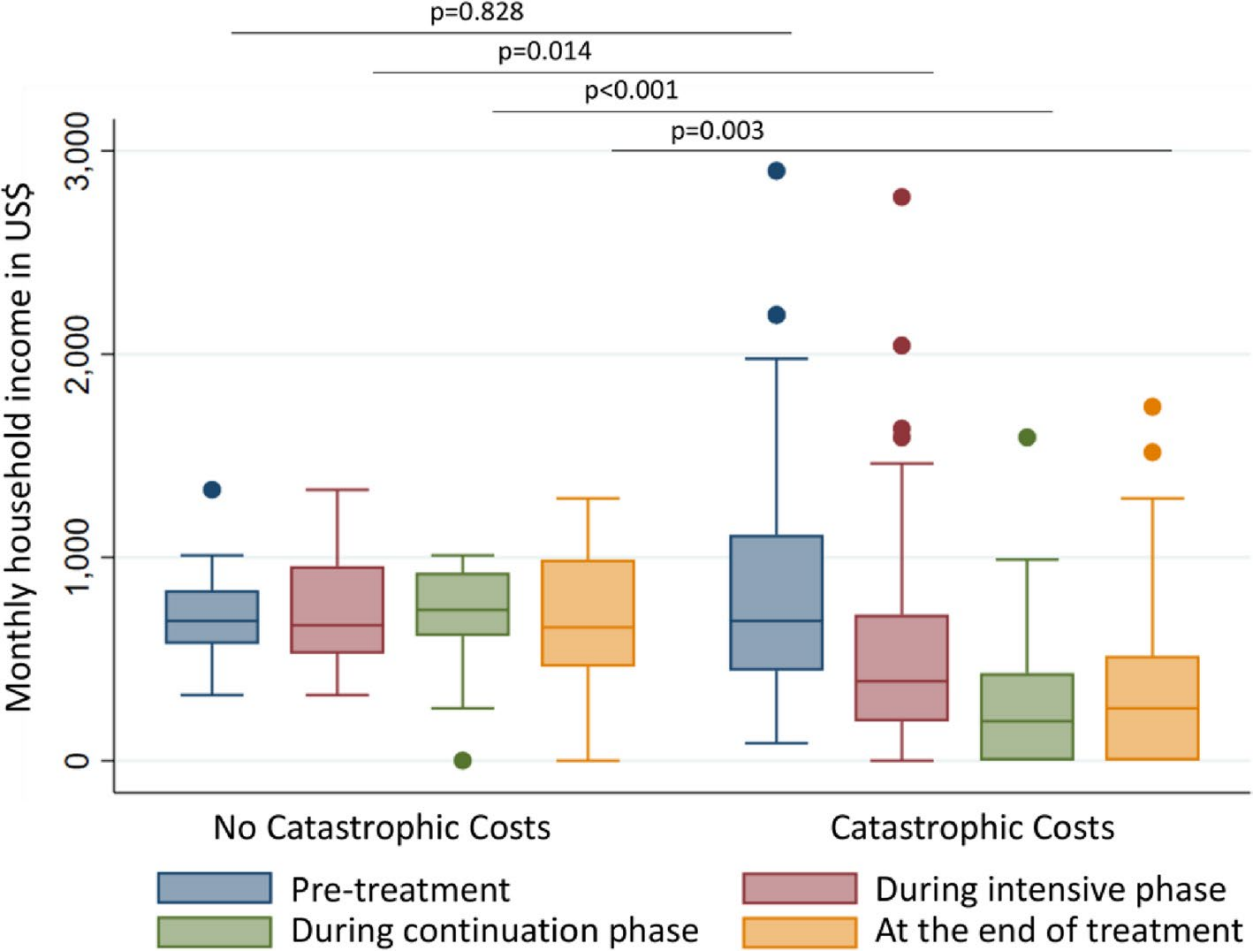
Variations in household income before, during and at the end of treatment by catastrophic costs

The pre-treatment median monthly household income was similar between the two groups. However, by the end of the treatment the monthly household income was significantly lower among people experiencing catastrophic costs (258 [IQR: 0–516] USD vs. 656 [IQR: 462–989] USD; *p* = 0.003) (Table 4). With the longitudinal study design, we observed a steady decline in household income among people experiencing catastrophic costs throughout the episode of TB with insufficient recovery to return to pre-TB income levels by the end of treatment (Fig. 3).

**Table 4:** Median household income before and during multidrug-resistant tuberculosis treatment by experiencing catastrophic costs

	No catastrophic costs (*N* = 12)	Catastrophic costs (*N* = 81)	Total (*N* = 93)	*p*-value^*^
	Median (IQR)	Median (IQR)	Median (IQR)	
Pre-treatment (per month)				
Self-Reported household income in USD	688.0 (574.1–838.5)	688.0 (442.9–1110.7)	688.0 (516–1044.9)	0.828
Intensive Phase (per month)				
Self-Reported household income in USD	666.5 (526.8–956.8)	391.3 (193.5–718.1)	451.5 (236.5–774.0)	0.014
Continuation Phase (per month)				
Self-Reported household income in USD	741.8 (613.8–924.5)	193.5 (0.0–430.0)	236.5 (25.8–559.0)	< 0.001
End of Treatment (per month)				
Self-Reported household income in USD	655.8 (462.3–989.0)	258.0 (0.0–516.0)	365.5 (0.0–636.4)	0.003

Estimation of catastrophic costs using the output approach at a 20% threshold

*Abbreviation*: *IQR* Interquartile range

^*^Wilcoxon rank sum test

**Fig. 4:**
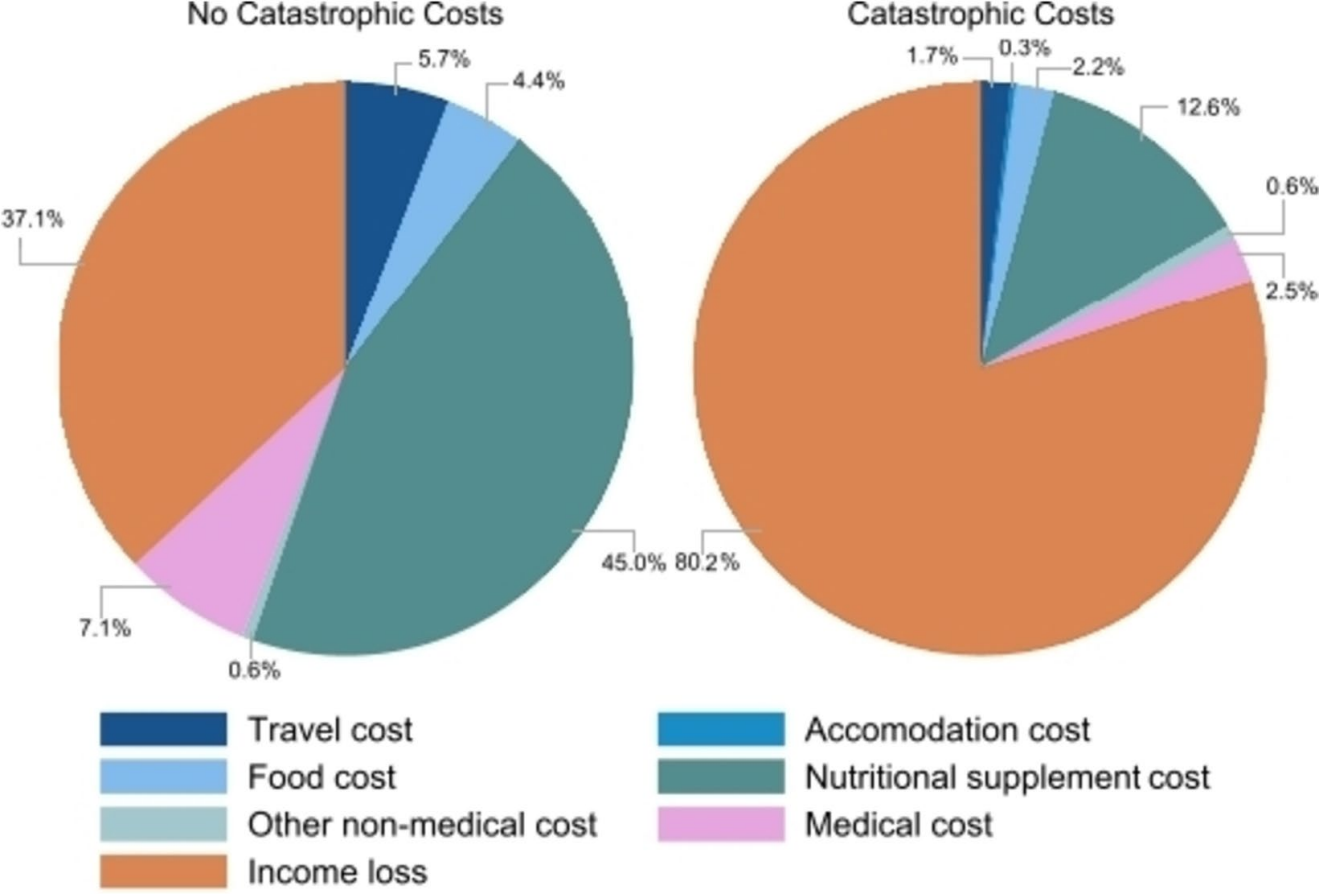
Breakdown of total costs into income loss (orange), direct non-medical (blue-shaded) and medical (purple) costs during the entire treatment

During the entire treatment period, total median costs were significantly higher among people with MDR-TB who experienced catastrophic costs (4003 [IQR: 2650–6537] USD vs. 1044 [IQR: 1044–1353] USD; *p* < 0.001) driven by higher median household income loss (2838 [IQR: 1548–5418] USD vs. 301 [IQR: 0–824] USD; *p* < 0.001) compared to people with MDR-TB who did not experience catastrophic costs (Table 5). Amongst people with MDR-TB who experienced catastrophic costs, household income loss contributed the largest proportion of total costs (80%), followed by non-medical costs (17%) (Fig. 4). Median total non-medical costs were 681 [IQR: 396–1161] USD, with the largest proportion from nutritional supplements (74% of non-medical costs; 461 [IQR: 233–810] USD), then food (12%; 44 [IQR: 4–146] USD) and travel (10%; 60 [IQR: 36–92] USD) (Table 5, Additional file 2, Fig. S2). Around half of the total non-medical costs were incurred during the intensive phase (367 [IQR: 184–664] USD), which then declined during the remainder of treatment (Additional file 1, Table S2). Direct medical costs were highest prior to treatment but, overall, contributed the lowest share of total costs at around 3% during the entire treatment (49 [IQR: 9–142] USD) (Table 5; Additional fil 2, Fig. S2).

**Table 5:** Median direct and indirect costs before and during multidrug-resistant tuberculosis treatment by experiencing catastrophic costs

	No catastrophic costs (*N* = 12)	Catastrophic costs (*N* = 81)	Total (*N* = 93)	*p*-value^*^
	Median (IQR)	Median (IQR)	Median (IQR)	
Pre-treatment				
Direct Medical Cost in USD				
Total medical (including radiography, TB medicine etc.)	34.0 (13.1–130.7)	83.59 (27.0–188.6)	75.8 (25.2–177.6)	0.220
Direct Non-Medical Cost in USD				
Total non-medical	11.2 (7.1–34.5)	21.5 (7.7–61.7)	21.4 (7.7–60.0)	0.547
Indirect Cost in USD				
Hours lost due to ambulatory care visits	21.6 (9.3–63.5)	30.0 (16.3–64.5)	29.9 (15.5–64.5)	0.276
Hours lost*hourly wage	27.4 (16.2–93.9)	39.8 (24.8–100.1)	39.1 (24.8–100.1)	0.302
Total Cost in USD				
Sum of direct medical, and non-medical cost	111.3 (21.5–147.3)	114.3 (38.8–225.8)	113.1 (37.5–224.1)	0.308
During entire treatment				
Direct Medical Cost in USD				
Total medical (including radiography, TB medicine etc.)	24.3 (3.0–129.3)	51.5 (10.1–146.6)	49.0 (9.4–141.5)	0.448
Direct Non-Medical Cost in USD				
Travel	66.7 (42.5- 96.8)	60.1 (35.7–89.1)	60.2 (36.0–91.7)	0.748
Accommodation^a^	0.0 (0.0–0.0)	0.0 (0.0–0.0)	0.0 (0.0–0.0)	0.500
Food	20.3 (0.0–58.6)	53.8 (3.9–162.6)	43.9 (3.9–145.6)	0.098
Nutritional Supplements^a^	570.0 (193.4–738.0)	456.2 (235.9–821.6)	460.8 (232.6–809.9)	0.973
Other non-medical	0.0 (0.0–4.0)	0.0 (0.0–8.9)	0.0 (0.0–5.6)	0.971
Total non-medical	756.5 (245.0–906.0)	665.0 (405.3–1226.4)	680.7 (396.0–1161.4)	0.409
Indirect Cost in USD				
Hours lost due to ambulatory care visits	103.0 (62.8–158.9)	138.7 (80.0–214.8)	138.1 (75.1–208.9)	0.114
Hours lost times hourly wage	155.4 (46.8–317.1)	227.0 (95.6–398.7)	214.3 (89.5–397.3)	0.1800
Household income lost in USD	301.0 (0.0–823.5)	2838.0 (1548.0–5418.0)	2653.1 (946.0–5224.5)	< 0.001
Total Cost in USD				
Sum of direct medical, non-medical and income loss	1043.7 (1043.7–1352.9)	4002.8 (2650.3–6536.7)	3312.5 (1831.5–5949.6)	< 0.001

Estimation of catastrophic costs using the output approach at a 20% threshold

*Abbreviations: TB* Tuberculosis, *IQR* Interquartile range

^*^Wilcoxon rank sum test

^a^Contains in total six missing values imputed using the median

### Risk factors of catastrophic costs

In univariable logistic regression analysis, lower educational attainment, unemployment, and poorer wealth were associated with experiencing catastrophic costs (Table 6). In the final multivariable regression analysis, becoming unemployed at the beginning of the treatment (aOR = 35.6 [95% CI: 2.7–470.3]; *p* = 0.007), having a lower (primary or formal) educational level (aOR = 22.3 [95% CI: 1.5–344.1]; *p* = 0.026), and being the primary income earner of the household (aOR = 11.2 [95% CI: 1.6–80.5]; *p* = 0.016) were independently associated with experiencing catastrophic costs (Table 6). Having HIV, experiencing social exclusion, or belonging to the poorest wealth tercile were perfect predictors of experiencing catastrophic costs, thus were not included or replaced by the wealth index in the final model.

**Table 6:** Univariable and multivariable logistic regression with factors associated with experiencing catastrophic costs

Characteristics	Univariable Logistic Regression		Multivariable Logistic Regression	
	OR [95% CI]	*p*-value	aOR [95% CI]	*p*-value
**Individual and household characteristics**				
Age				
Years of age	1.00 [0.96–1.04]	0.849	0.97 [0.91–1.02]	0.261
Gender				
Male	2.50 [0.71–8.83]	0.155	4.97 [0.80–30.98]	0.086
Participant’s education level				
Primary school and below	6.25 [1.29–30.35]	**0.023**	22.32 [1.45–344.09]	**0.026**
Completed secondary school and above	(reference)		(reference)	
Household status				
Primary income earner before disease	4.89 [1.01–23.67]	**0.049**	11.23 [1.57–80.46]	**0.016**
Household wealth				
Wealth index	0.58 [0.39–0.85]	**0.006**	0.70 [0.41–1.19]	0.165
**Socioeconomic impact of MDR-TB treatment**				
Employment status during treatment				
Change to unemployment at the beginning of treatment	13.08 [1.61–106.12]	**0.016**	35.58 [2.69–470.27]	**0.007**

Estimation of catastrophic costs using the output approach at a 20% threshold

*Abbreviations*: *MDR-TB* Multidrug-resistant tuberculosis, *CI* Confidence interval, *OR* Crude odds ratio, *aOR* Adjusted odds ratio

### Sensitivity analysis

The proportion of people experiencing catastrophic costs at a 20% threshold differed across a range of different approaches (Table 7). Using the output approach with income loss and excluding amounts of vouchers and other social protection mechanisms resulted in an increase of one percentage point in catastrophic costs prevalence (88.2% [95% CI: 79.8–93.4]). In contrast, when using the output approach and asset-based income as an indicator of the household’s capacity to pay, catastrophic costs prevalence was 77.4% (95% CI: 67.7–84.8). Using the human capital approach, the proportion of people experiencing catastrophic costs was almost four-times lower compared to the output approach (23.7% [95% CI: 16.0–33.4] vs 87.1% [95%CI: 78.5–92.6]). Including only direct (medical and non-medical) costs in the estimation would amount to 15.1% (95% CI: 9.0–24.0) of people experiencing catastrophic costs.

**Table 7:** Sensitivity analysis showing the proportion of households experiencing catastrophic costs at various thresholds and approaches

Catastrophic costs thresholds	Primary analysis^a^ in % [95% CI]	Alternative I^b^ in % [95% CI]	Alternative II^b^ in % [95% CI]	Alternative III^b^ in % [95% CI]	Alternative IV^b^ in % [95% CI]
1	100.00 [-]	100.00 [-]	100.00 [-]	100.00 [-]	97.85 [91.67–99.47]
2	100.00 [-]	100.00 [-]	100.00 [-]	97.85 [91.67–99.47]	96.77 [90.34–98.97]
4	100.00 [-]	100.00 [-]	98.92 [92.59–99.85]	90.32 [82.30–94.93]	78.49 [68.86–85.77]
10	97.85 [91.67–99.47]	97.85 [91.67–99.47]	89.25 [81.02–94.16]	54.76 [43.48–63.74]	37.63 [28.29–48.00]
20	87.10 [78.50–92.58]	88.17 [79.76–93.38]	77.42 [67.69–84.87]	23.66 [16.03–33.47]	15.05 [9.06–23.97]
30	77.42 [67.69–84.87]	78.49 [68.86–85.77]	59.14 [48.77–68.76]	13.98 [8.23–22.74]	11.83 [6.62–20.24]
40	58.06 [47.70–67.76]	60.22 [49.84–69.75]	49.46 [39.33–59.64]	11.83 [6.62–20.24]	7.53 [3.59–15.09]
50	33.33 [24.41–43.62]	43.01 [32.24–53.36]	35.48 [26.34–45.82]	6.45 [2.90–13.75]	6.45 [2.90–13.75]
60	16.13 [9.89–25.19]	18.28 [11.61–27.59]	22.58 [15.13–32.31]	4.30 [1.60–11.03]	3.23 [1.03–9.66]
100	2.15 [0.53–8.33]	3.23 [1.03–9.66]	6.45 [2.89–13.75]	2.15 [0.53–8.33]	0.00 [-]
200	0.00 [-]	0.00 [-]	0.00 [-]	0.00 [-]	0.00 [-]

*Abbreviation:* *CI *Confidence interval

^a^Primary analysis uses the output approach including amount of vouchers and social welfare payments or cash transfers as income

^b^Alternative analysis: Alternative I uses the output approach excluding amount of vouchers and social welfare payments or cash transfers; Alternative II uses the output approach with asset-based income instead of self-reported income; Alternative III uses the human capital approach; Alternative IV uses direct costs only

**Fig. 5:**
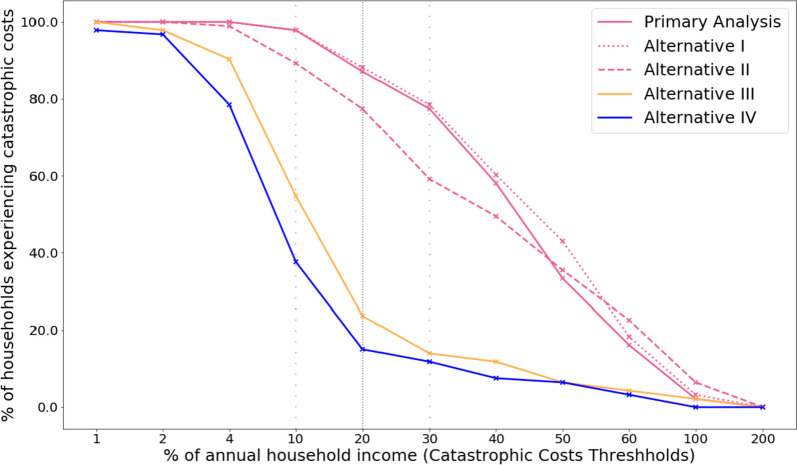
Sensitivity analysis of catastrophic costs prevalence at different thresholds (from 1-200%) using different estimation approaches. Legend: Primary analysis uses the output approach including amount of vouchers and social welfare payments or cash transfers as income. Alternative I uses the output approach excluding amount of vouchers and social welfare payments or cash transfers. Alternative II uses the output approach with asset-based income instead of self-reported income. Alternative III uses the human capital approach. Alternative IV uses direct costs only

At different thresholds using the primary approach, we observed a decrease in catastrophic costs prevalence to 77.4% (95% CI: 67.7–84.9) and 33.3% (95% CI: 24.4–43.6) at a threshold of 30% and 50% of annual income, respectively (Table 7, Fig. 5). At the 30% and 50% thresholds, becoming unemployed at the beginning of the treatment remains independently associated with experiencing catastrophic costs (aOR = 10.0 [95% CI: 3.2–31.1]; *p* < 0.001) (Additional file 1, Table S3).

## Discussion

This study showed that catastrophic costs are common among people with MDR-TB and their households and that the main contributors are income loss and direct non-medical costs. Direct non-medical costs were highest during the intensive treatment phase, predominantly due to nutritional supplements, food and travel, yet vouchers to cover these costs were more commonly reported as being received towards the end of the treatment. People with MDR-TB who experienced catastrophic costs faced a greater decline of household income during MDR-TB treatment than those who did not experience catastrophic costs, and throughout the duration of their treatment, income did not return to pre-TB levels. Job loss at the beginning of the treatment, lower educational level, and being the primary income earner of the household were identified as risk factors for catastrophic costs incurrence.

The proportion of people experiencing catastrophic costs among MDR-TB-affected households in HCMC, Viet Nam (87% [95% CI: 79–93) is similar to estimates from other lower middle-income countries in Southeast Asia [13, 17, 40] and to the global average proportion of DR-TB-affected households experiencing catastrophic costs (87% [95% CI: 80–93%]) [3]. Compared to national estimates for Viet Nam in 2016, the catastrophic costs prevalence of our study is lower (98% vs. 87%) and, instead of direct non-medical costs in 2016, income loss was identified as the main cost contributor (32% vs. 80% household income loss, 46% vs. 17% direct non-medical cost) [12]. These differences may potentially be accounted for due to methodological and geographical differences between our subnational study and the 2016 national PCS. The national PCS took a cross-sectional rather than longitudinal approach to estimate catastrophic costs, featured study participants on a 20-month rather than a 9-month regimen, and recruited nationally in predominantly secondary cities and rural areas. Our study was longitudinal, focused on people with MDR-TB being treated with shorter regimens and only recruited in HCMC, which has relatively high monthly average income per capita, population growth rate, and living costs [21].

In our study, direct medical costs contributed the smallest share of the total costs during MDR-TB treatment. However, before MDR-TB treatment, medical costs were around four-times higher than non-medical costs. Similar observations have been made in Myanmar [41], Lao People’s Democratic Republic [13], and the Philippines [15], although the nominal amount of medical costs during MDR-TB treatment were higher in those countries compared to our results. A possible explanation is that following treatment initiation, medical costs are minimized due to free TB tests, services implemented as part of the NTP and fewer hospitalisations. Viet Nam has a remarkably high Universal Health Coverage (UHC) service coverage index among lower-middle-income countries [3]. However, Viet Nam has one of the highest rates of catastrophic costs incurrence among people with TB in the world [3]. Thus, in Viet Nam, efforts are needed to mitigate especially direct non-medical costs and income loss not included in SHI coverage.

People not covered by SHI need to be supported with additional social protection programmes. Results from the first national TB PCS have triggered a series of changes in policy action and practice showing the government’s commitment and efforts to reduce catastrophic costs such as the development of packages for ambulatory TB services advocating for SHI coverage, or the creation of national policy guide on interventions to reduce treatment costs [42]. Our results emphasize the need to further support people with MDR-TB by addressing specific social protection elements that are involved in higher treatment costs compared to DS-TB.

This could potentially be achieved using an approach that incorporated three dimensions. Firstly, by increasing monetary transfers and travel or food support. Secondly, by building a more people-centred, decentralised delivery system for TB care services that minimizes hospitalisation and time lost due to care seeking. Thirdly, by expanding linkage to other TB-related services such as quality assured MDR-TB diagnosis and treatment services [43]. The latter includes the provision and prioritization of the less costly shorter treatment regimen as recommended by the WHO over the 20-month regimen [10, 11]. Social protection mechanisms to cover income loss and direct non-medical costs have the potential to reduce catastrophic costs prevalence, as shown in India and South Africa [44, 45] as well as various European countries [46]. Implementing social protection mechanisms that are complementary to efforts towards UHC in Viet Nam could act synergistically to eliminate catastrophic costs.

Although the majority of participants (93%) in this study received some form of social support during their treatment, this support had a weak effect in lowering catastrophic costs. Several factors and misconceptions might have prevented greater reduction of catastrophic costs despite the presence of social support. Participants may be hesitant in using free services potentially due to doubts about their quality of care compared to paid services [47]. Furthermore, the time at which the affected person received the social support might also influence the protective effect. For example, total costs and, in particular, non-medical costs seem to be highest at the beginning of the treatment. Therefore, even if social support such as vouchers were received later during the treatment it would not cover earlier financial shock. Addressing misconceptions around quality of subsidized services and the provision of timely support for MDR-TB affected households are needed to improve social protection mechanisms.

On the other hand, catastrophic costs as a binary indicator only provides information on whether the TB affected households experienced financial shock due to their treatment and if they are at risk of adverse TB outcome [4]. It does not represent impoverishment and the size of the poverty gap created due to the treatment for which a more in-depth analysis would be necessary. Thus, by focusing merely on the catastrophic costs indicator as outcome may lead to an underestimation of positive effects created by existing social protection mechanisms in preventing impoverishment, covering costs for TB services, or improving productivity and the household’s capacity to pay.

People experiencing catastrophic costs in this study had to deal with a strong decline in household income with weak signs of recovery towards the end of the treatment, yet without reaching pre-TB household income levels. In contrast, a study in HCMC with similar longitudinal adaption of the PCS found a near-full recovery during the continuation phase among people with DS-TB and a comparatively lower income loss during the treatment compared to our study [31]. This suggests that people with MDR-TB experience a greater financial shock during the treatment from which they recover more slowly compared to DS-TB. About half of our study population were affected by a previous episode of TB or had another household member affected by TB. Missing recovery from financial hardships experienced during previous TB episodes may have contributed to a greater income loss. Consequently, MDR-TB affected households may require greater efforts to compensate for the income lost for example by returning to coping strategies such as taking loans or selling assets. The recovery from income loss may also depend on additional activities from household members, or the ability of the MDR-TB affected person to keep or regain their employment status from before the treatment. To support this process, sickness insurance, disability grants and job protection could be provided, or their coverage expanded to people and households with MDR-TB.

The risk factors identified in this study were similar to risk factors reported in other research studies [13, 14, 17, 48]. Apart from job protection and financial support, educational support interventions and integration into existing social support mechanisms could be provided for people at risk so that they are enabled to seek and continue care as well as to make informed choices about their own health [49].

Furthermore, all the participants who self-reported experiencing stigma also experienced catastrophic costs. Thus, further research with increased sample size is needed to study a potential association between stigma among people with MDR-TB and their experience of catastrophic costs within the country setting. In Viet Nam, stigma and self-isolation among people with DR-TB are connected and shaped by cultural expectations [50]. Consequences include reduced psychological and social wellbeing which may affect the working and productivity state of the affected person and household, treatment adherence with the risk of MDR-TB transmission and adverse treatment outcome [37]. Social protection packages and the NTP might want to consider psychosocial support and counselling services within the cultural context and understanding of MDR-TB as also suggested by Smith et al. [51].

Other risk factors supported by literature yet not found to be associated with catastrophic costs incurrence in our study were previous TB episodes, hospitalisation, BMI, and treatment delay [4, 14, 17, 52]. Moreover, there was a lack of evidence in the literature regarding the impact of coping strategies and social support as potential predictors of experiencing catastrophic costs. The use of coping strategies and the receipt of social support seem to be more frequent among people experiencing catastrophic costs in this study, yet due to a small sample size and the lack of statistical evidence these study variables were excluded in the final model. Besides sample size limitations, deviations from literature findings could also be explained by different study populations. Whereas other studies usually pool together people with DS-TB and MDR-TB before analysing associations with catastrophic costs [4, 14, 40], our study population was explicitly addressing people with MDR-TB only. People with MDR-TB could acquire resistance from a previous TB episode [39]. In this case, the person was already exposed to TB care facilities, treatment procedures and support services which may have facilitated the process of seeking and receiving care or social support thus for example lowering the risk of catastrophic costs due to hospitalisations.

### Study limitations

This study has various limitations. Due to the small sample size, low frequency of observations within the group of people not experiencing catastrophic costs caused numerical issues during logistic regression analyses and hence wide confidence intervals of odds ratios [53]. Future studies may need to consider increasing the sample size or pooling data of low frequency observations to explore their association with catastrophic costs in the Vietnamese setting with greater confidence, and to examine the potential role of social protection in this vulnerable group.

Secondly, we used the output approach for the analysis of risk factors which may be error-prone in settings without a large formal employment sector due to the unreliability of self-reported income [35]. As a considerable proportion (45%) of our study population belonged to the informal sector, we need to be aware of potential social desirability bias in self-reporting of income during the interview. To a certain extent, the longitudinal design controls for recall bias within each treatment phase while the risk of under- or overestimation of income remains.

Thirdly, the output approach does not distinguish between household income loss due to reasons unrelated to TB care-seeking. HCMC experienced several partial and complete lockdowns due to COVID-19 during the study and participants’ treatment period [33]. As a consequence, household income across the entire welfare distribution declined which might affected the households’ capacity to recover from financial shock. Farmers for example needed to drastically lower selling prices of their products. Additional trading restrictions and prise increase of consumables indicate decreasing income and increasing costs due to the pandemic [7]. Moreover, a greater gender gap in unemployment for females was created [54]. Thus, this study potentially underestimates the impact of risk factors associated with experiencing catastrophic costs during the pandemic among females. In this context, the outbreak has introduced additional unmeasured or unexplored associations that could have potentially confounded the income loss due to TB treatment.

Fourthly, we excluded 24 participants from the final analysis due to loss of follow up or other reasons which may have introduced a bias as the participants excluded were significantly older and had a lower wealth status.

### Generalizability and transferability

This study is restricted to a specific group of people receiving MDR-TB treatment with a nine-month regimen living in HCMC which is the largest city in Viet Nam with an average monthly income per capita above the country average and which at this time was severely affected by the COVID-19 outbreak. The difference is reflected when comparing the average monthly household income before treatment of this study cohort to national estimates (US$ 807 vs. 368) [12]. With higher hourly wage, income from labour activities increased which affected the household’s capacity to pay. As this analysis focused on people within the nine-months regimen, it is not generalizable to people who received MDR-TB treatment to alternative regimens such as the 20-months regimens. People who received a 20-months regimen suffer from over 40% higher total treatment costs compared to those in the nine-months regimen [11]. Furthermore, of the 172 eligible people, only 94 were included in this analysis; thus, the cohort may not represent the majority of people living in HCMC and receiving the nine-months regimen.

The study also does not cover the population groups outside of or in rural Viet Nam such as ethnic minorities in mountainous regions. Those areas may be exposed to greater access barriers when seeking care at health facilities and restricted in the efficiency of service delivery due to limited resources and health workforce. Consequently, catastrophic costs among the population group in those areas could be expected to be higher due to greater time and productivity loss. Although common risk factors have been found in different country contexts, the results are specific to the Vietnamese healthcare and delivery system.

All study participants were enrolled in the NTP which offers a standardized package of treatment and care services. People with MDR-TB who are not enrolled in the NTP may not have access to the same service levels which could result in prolonged treatment, greater costs, and additional risk factors to experience catastrophic costs.

## Conclusion

This study found that catastrophic costs incurrence among people with MDR-TB and their household living in HCMC remains high and a challenge towards reaching the goal of eliminating catastrophic costs as part of the SDG 3.8, 1.3 and the WHO End TB Strategy. Total costs during the treatment were driven primarily by income loss and direct non-medical cost. Excluding the amounts received by social protection mechanisms led to an increase in the proportion of catastrophic costs. Social protection thus seems to be effective in alleviating indirect and direct non-medical costs; however, not yet sufficient to protect the majority of MDR-TB affected households from experiencing catastrophic costs. Thus, expansion or revision of social protection packages are needed to identify and support the vulnerable population early on in their treatment. Being the primary income earner of the household before TB illness and becoming unemployed at the beginning of the treatment were more likely to incur catastrophic costs and thus in need of those social protection packages.

### Supplementary Information

**Additional file 1:  Table S1. **The Strengthening the Reporting of Observational Studies in Epidemiology (STROBE) Statement: checklist for cohort studies.** Table S2.** Median costs due to multidrug-resistant tuberculosis treatment by experiencing catastrophic costs during different treatment phases. **Table S3.** Univariable and multivariable logistic regression with factors associated with experiencing catastrophic costs using alternative thresholds.

**Additional file 2: Figure S1. **Percentage of people receiving any social welfare payments/ cash transfers (A) or vouchers (B). **Figure S2.** Breakdown of total costs (A) and direct non-medical costs (B) for entire study population. **Figure S3.** Conceptual Framework guiding the analysis of risk factors (purple) associated with the outcome of catastrophic costs (blue) and potential confounders (yellow).

**Additional file 3. **Patient cost survey for the first interview timepoint.

**Additional file 4. **Patient cost survey for the second and third interview timepoint.

**Additional file 5. **Description of alternative estimation approaches of catastrophic costs.

## Data Availability

Upon reasonable request and with permission from the Pham Ngoc Thach Provincial TB Hospital’s Institutional Review Board, data and statistical analysis files can be made available from RF. Restrictions apply to the availability of these data because they contain information that could compromise the privacy of research including clinical patient information, and thus are not being made publicly available.

## Abbreviations

aOR: Adjusted odds ratio
BMI: Body mass index
CI: Confidence interval
cOR: Crudes odds ratio
COVID-19: Coronavirus Disease 2019
DOT: Directly observed therapy
DS-TB: Drug-susceptible tuberculosis
GDP: Gross-domestic product
HCMC: Ho Chi Minh City
HIV: Human immunodeficiency virus
IQR: Interquartile range
MDR-TB: Multidrug-resistant tuberculosis
NGO: Non-governmental organisation
NTP: National tuberculosis programme
PCS: Patient cost survey
pDST: Phenotypic drug susceptibility testing
PMDT: Programmatic management of drug-resistant tuberculosis
PNT: Pham Ngoc Thach hospital
SD: Standard deviation
SHI: Social health insurance
TB: Tuberculosis
UHC: Universal health coverage
USD: United states dollar
VND: Vietnamese dong
WHO: World Health Organization
