# Supplementary material for: Determinants of catastrophic costs among households affected by multi-drug resistant tuberculosis in Ho Chi Minh City, Viet Nam: a prospective cohort study
Source: BMC Public Health. 2023 Dec 3;23:2372. doi: 10.1186/s12889-023-17078-5 (PMC10693707; doi:10.1186/s12889-023-17078-5)
Supplement: Supplementary file 1 — Additional file 1: Table S1. The Strengthening the Reporting of Observational Studies in Epidemiology (STROBE) Statement: checklist for cohort studies. Table S2. Median costs due to multidrug-resistant tuberculosis treatment by experiencing catastrophic costs during different treatment phases. Table S3. Univariable and multivariable logistic regression with factors associated with experiencing catastrophic costs using alternative thresholds. [file 12889_2023_17078_MOESM1_ESM.pdf]

SUPPLEMENTARIES

Table S1 The Strengthening the Reporting of Observational Studies in Epidemiology (STROBE) Statement: checklist for cohort studies

	Item No	Recommendation	Page No
**Title and abstract**	1	(*a*) Indicate the study's design with a commonly used term in the title or the abstract	1
		(*b*) Provide in the abstract an informative and balanced summary of what was done and what was found	1-2
**Introduction**			
Background/rationale	2	Explain the scientific background and rationale for the investigation being reported	2
Objectives	3	State specific objectives, including any prespecified hypotheses	2
**Methods**			
Study design	4	Present key elements of study design early in the paper	2-3
Setting	5	Describe the setting, locations, and relevant dates, including periods of recruitment, exposure, follow-up, and data collection	3-4
Participants	6	(a) Give the eligibility criteria, and the sources and methods of selection of participants. Describe methods of follow-up	4-5
		(b) For matched studies, give matching criteria and number of exposed and unexposed	n/a
Variables	7	Clearly define all outcomes, exposures, predictors, potential confounders, and effect modifiers. Give diagnostic criteria, if applicable	4-6
Data sources/ measurement	8*	For each variable of interest, give sources of data and details of methods of assessment (measurement). Describe comparability of assessment methods if there is more than one group	4-6
Bias	9	Describe any efforts to address potential sources of bias	6, 16
Study size	10	Explain how the study size was arrived at	4
Quantitative variables	11	Explain how quantitative variables were handled in the analyses. If applicable, describe which groupings were chosen and why	4-6
Statistical methods	12	(*a*) Describe all statistical methods, including those used to control for confounding	6
		(*b*) Describe any methods used to examine subgroups and interactions	6
		(*c*) Explain how missing data were addressed	5-6
		(*d*) If applicable, explain how loss to follow-up was addressed	n/a
		(*e*) Describe any sensitivity analyses	5, additional file 5
**Results**			
Participants	13*	(a) Report numbers of individuals at each stage of study—eg numbers potentially eligible, examined for eligibility, confirmed eligible, included in the study, completing follow-up, and analysed	6-7
		(b) Give reasons for non-participation at each stage	6-7
		(c) Consider use of a flow diagram	6
Descriptive data	14*	(a) Give characteristics of study participants (eg demographic, clinical, social) and information on exposures and potential confounders	8-9
		(b) Indicate number of participants with missing data for each variable of interest	11
		(c) Summarise follow-up time (eg, average and total amount)	4-5, 8
Outcome data	15*	Report numbers of outcome events or summary measures over time	7-8
Main results	16	(a) Give unadjusted estimates and, if applicable, confounder-adjusted estimates and their precision (eg, 95% confidence interval). Make clear which confounders were adjusted for and why they were included	6, 12
		(b) Report category boundaries when continuous variables were categorized	n/a
		(c) If relevant, consider translating estimates of relative risk into absolute risk for a meaningful time period	n/a
Other analyses	17	Report other analyses done—eg analyses of subgroups and interactions, and sensitivity analyses	13-14
**Discussion**			
Key results	18	Summarise key results with reference to study objectives	13-14
Limitations	19	Discuss limitations of the study, taking into account sources of potential bias or imprecision. Discuss both direction and magnitude of any potential bias	16

Interpretation	20	Give a cautious overall interpretation of results considering objectives, limitations, multiplicity of analyses, results from similar studies, and other relevant evidence	13-16
Generalisability	21	Discuss the generalisability (external validity) of the study results	16-17
**Other information**			
Funding	22	Give the source of funding and the role of the funders for the present study and, if applicable, for the original study on which the present article is based	17

*Give information separately for exposed and unexposed groups.

Note: An Explanation and Elaboration article discusses each checklist item and gives methodological background and published examples of transparent reporting. The STROBE checklist is best used in conjunction with this article (freely available on the Web sites of PLoS Medicine at http://www.plosmedicine.org/, Annals of Internal Medicine at http://www.annals.org/, and Epidemiology at http://www.epidem.com/). Information on the STROBE Initiative is available at http://www.strobe-statement.org.

Table S2 Median costs due to multidrug-resistant tuberculosis treatment by experiencing catastrophic costs during different treatment phases

	**No catastrophic costs (N=12)** Median (IQR)	**Catastrophic costs (N=81)** Median (IQR)	**Total (N=93)** Median (IQR)	**p-value** [a]
**Intensive Phase**				
Direct Medical Cost				
Total medical (including radiography, TB medicine etc.)	1.5 (0.0-4.1)	1.0 (0.0-24.0)	1.0 (0.0-21.2)	0.518
Direct Non-Medical Cost				
Travel	47.8 (35.4-72.0)	44.7 (28.4-69.5)	45.3 (30.6-69.5)	0.688
Accommodation*	0.0 (0.0-0.00)	0.0 (0.0-0.0)	0.0 (0.0-0.0)	0.584
Food	4.1 (0.0-41.9)	30.1 (0.0-113.2)	28.6 (0.0-111.7)	0.266
Nutritional supplements*	235.0 (62.6-372.4)	256.9 (111.7-391.0)	256.9 (111.7-391.0)	0.801
Other non-medical	0.0 (0.0-2.2)	0.0 (0.0-0.0)	0.0 (0.0-0.0)	0.850
Total non-medical	315.8 (111.7-532.8)	367.1 (196.5-695.7)	367.1 (184.3-664.3)	0.276
Indirect Cost				
Hours lost due to ambulatory care visits	75.9 (43.5-129.8)	99.2 (56.3-159.3)	98.9 (53.4-155.9)	0.319
Hours lost times hourly wage	114.2 (32.0-198.4)	152.9 (65.9-307.6)	143.2 (63.8-276.4)	0.271
Household income lost in USD	0.0 (0.0-107.5)	1032.0 (154.8-1806.0)	774.0 (0.0-1548.0)	**<0.001**
Total Cost				
Sum of direct medical, non-medical and income loss	438.0 (276.4-534.2)	1521.8 (592.5-2436.9)	1314.7 (478.9-2040.7)	**<0.001**
**Continuation Phase (First Half)**				
Direct Medical Cost				
Total medical (including radiography, TB medicine etc.)	8.2 (0.0-58.7)	5.8 (0.0-75.3)	5.8 (0.0-64.5)	0.758
Direct Non-Medical Cost				
Travel costs	9.3 (4.9-12.9)	8.6 (4.5-15.5)	8.6 (4.5-15.1)	0.968
Accommodation	0.0 (0.0-0.0)	0.0 (0.0-0.0)	0.0 (0.0-0.0)	0.584
Food	0.6 (0.0-9.2)	1.3 (0.0-12.7)	1.3 (0.0-12.4)	0.694
Nutritional Supplements	107.1 (38.6-293.2)	100.5 (16.8-223.4)	100.5 (20.0-232.7)	0.494
Other non-medical	0.0 (0.0-0.0)	0.0 (0.0-0.0)	0.0 (0.0-0.0)	0.494
Total non-medical	169.8 (71.9-305.8)	128.5 (40.1-330.3)	128.5 (45.0- 330.3)	0.590
Indirect Cost				
Hours lost due to ambulatory care visits	8.7 (6.7-11.8)	13.7 (7.1-34.8)	12.3 (7.1-33.7)	0.203
Hours lost times hourly wage	12.4 (7.8-22.3)	24.4 (11.0-61.7)	22.2 (11.0-54.5)	0.142
Household income lost in USD	0.0 (0.0-860.0)	1038.0 (483.8-1916.7)	967.5 (258.0-1806.0)	**<0.001**
Total Cost				
Sum of direct medical, non-medical and income loss	356.3 (247.2-1124.7)	1279.4 (865.9-2186.4)	1212.6 (642.9-2079.3)	**0.002**
**Continuation Phase (Second Half)**				
Direct Medical Cost				
Total medical (including radiography, TB medicine etc.)	0.0 (0.0-17.0)	6.5 (0.0-49.0)	6.0 (0.0-46.9)	0.238
Direct Non-Medical Cost				
Travel costs	3.4 (1.6-4.6)	3.0 (1.4-5.2)	3.2 (1.4-4.9)	0.886

Accommodation	0.0 (0.0-0.0)	0.0 (0.0-0.0)	0.0 (0.0-0.0.)	0.700
Food	0.0 (0.0-1.5)	0.9 (0.00-7.5)	0.0 (0.0-6.9)	0.111
Nutritional Supplements	83.8 (26.1-167.8)	55.9 (0.0-162.9)	68.9 (0.0-162.9)	0.570
Other non-medical	0.0 (0.0-0.0)	0.0 (0.0-0.0)	0.0 (0.0-0.0)	-
Total non-medical	85.8 (26.7-171.2)	77.9 (12.9-175.3)	77.9 (12.9-175.3)	0.8028
Indirect Cost				
Hours lost due to ambulatory care visits	9.7 (3.8-15.2)	9.0 (4.5-15.2)	9.0 (4.5-15.2)	0.995
Hours lost times hourly wage	11.0 (4.6-23.2)	13.8 (5.3-29.0)	13.0 (5.3-29.0)	0.403
Household income lost in USD	80.6 (0.0-545.0)	1032.0 (215.0-1752.3)	774.0 (21.5-1677.0)	**0.006**
Total Cost				
Sum of direct medical, non-medical and income loss	215.4 (111.5-557.2)	1134.2 (352.5-1878.1)	1017.0 (291.9-1724.7)	**0.006**

[a] Wilcoxon rank sum test

* Contains in total six missing values imputed using the median

Abbreviations: HH – household, TB - tuberculosis

Table S3 Univariable and multivariable logistic regression with factors associated with experiencing catastrophic costs using alternative thresholds

Characteristics	**Primary analysis*,** 30% threshold				**Primary analysis*,** 50% threshold			
	**Univariable Logistic Regression**		**Multivariable Logistic Regression**		**Univariable Logistic Regression**		**Multivariable Logistic Regression**	
	cOR [95% CI]	p-value	aOR [95% CI]	p-value	cOR [95% CI]	p-value	aOR [95% CI]	p-value
**Individual and household characteristics**								
Age								
Years of age	1.00 [0.97-1.03]	0.811	1.01 [0.97-1.05]	0.533	1.01 [0.98-1.04]	0.354	1.04 [1.00-1.08]	0.076
Gender								
Male	1.29 [0.43-3.86]	0.717	1.29 [0.35-4.74]	0.705	0.71 [0.27-1.90]	0.498	0.49 [0.15-1.59]	0.235
Participant's education level								
Primary school or below	1.97 [0.72-5.32]	0.181	1.34 [0.31-5.80]	0.698	1.07 [0.45-2.52]	0.883	0.37 [0.12-1.24]	0.107
Completed secondary school and above	(reference)		(reference)		(reference)		(reference)	
Household status								
Primary income earner before disease	2.50 [0.87-7.16]	0.088	3.56 [1.00-12.68]	**0.050**	1.79 [0.75-4.29]	0.187	2.32 [0.81-6.62]	0.116
Household wealth								
Wealth index	0.73 [0.56-0.96]	**0.026**	0.81 [0.58-1.12]	0.200	0.82 [0.65-1.03]	0.084	0.76 [0.57-1.03]	0.074
**Socioeconomic impact of MDR-TB treatment**								
Employment status during treatment								
Change to unemployment at the beginning of treatment	14.09 [3.05-65.13]	**0.001**	18.10 [3.42-95.66]	**0.001**	6.69 [2.48-18.06]	**<0.001**	9.69 [3.09-30.37]	**<0.001**

*Primary analysis uses the output approach including amount of vouchers and social welfare payments or cash transfers as income
Abbreviations: MDR-TB – multidrug-resistant tuberculosis; CI – confidence interval; cOR – crude odds ratio; aOR – adjusted odds ratio
