# Supplementary material for: Determinants of catastrophic costs among households affected by multi-drug resistant tuberculosis in Ho Chi Minh City, Viet Nam: a prospective cohort study
Source: BMC Public Health. 2023 Dec 3;23:2372. doi: 10.1186/s12889-023-17078-5 (PMC10693707; doi:10.1186/s12889-023-17078-5)
Supplement: Supplementary file 2 — Additional file 2: Figure S1. Percentage of people receiving any social welfare payments/ cash transfers (A) or vouchers (B). Figure S2. Breakdown of total costs (A) and direct non-medical costs (B) for entire study population. Figure S3. Conceptual Framework guiding the analysis of risk factors (purple) associated with the outcome of catastrophic costs (blue) and potential confounders (yellow). [file 12889_2023_17078_MOESM2_ESM.pdf]

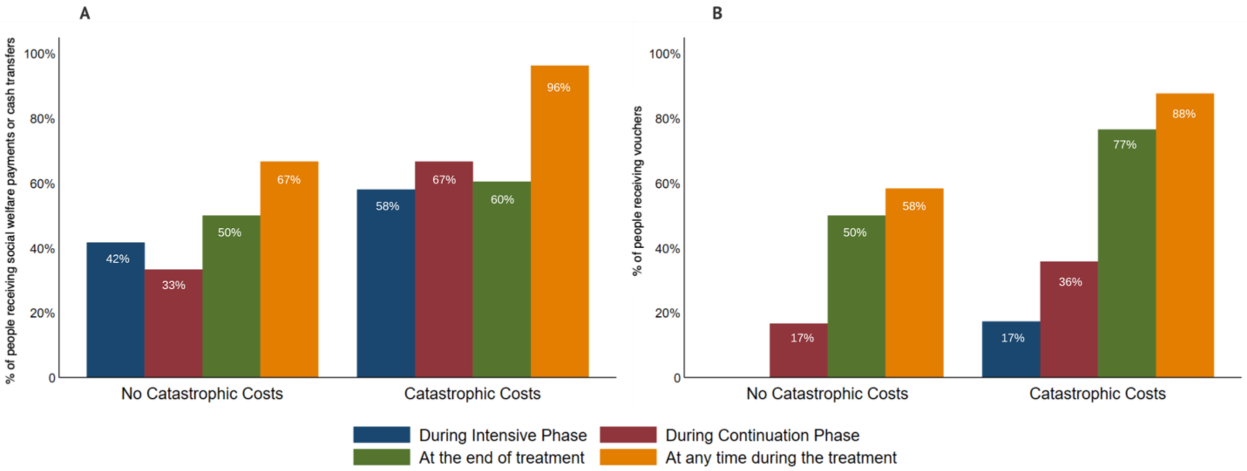

Figure S1 ***Percentage of people receiving any social welfare payments/ cash transfers (A) or vouchers (B)***

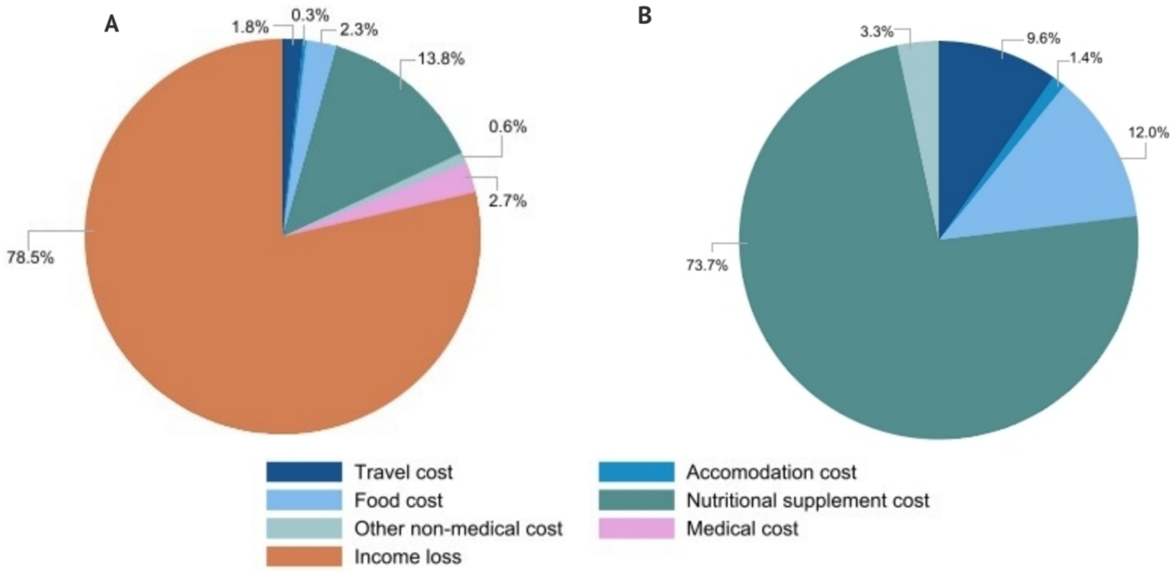

Figure S2 ***Breakdown of total costs (A) and direct non-medical costs (B) for entire study population.***

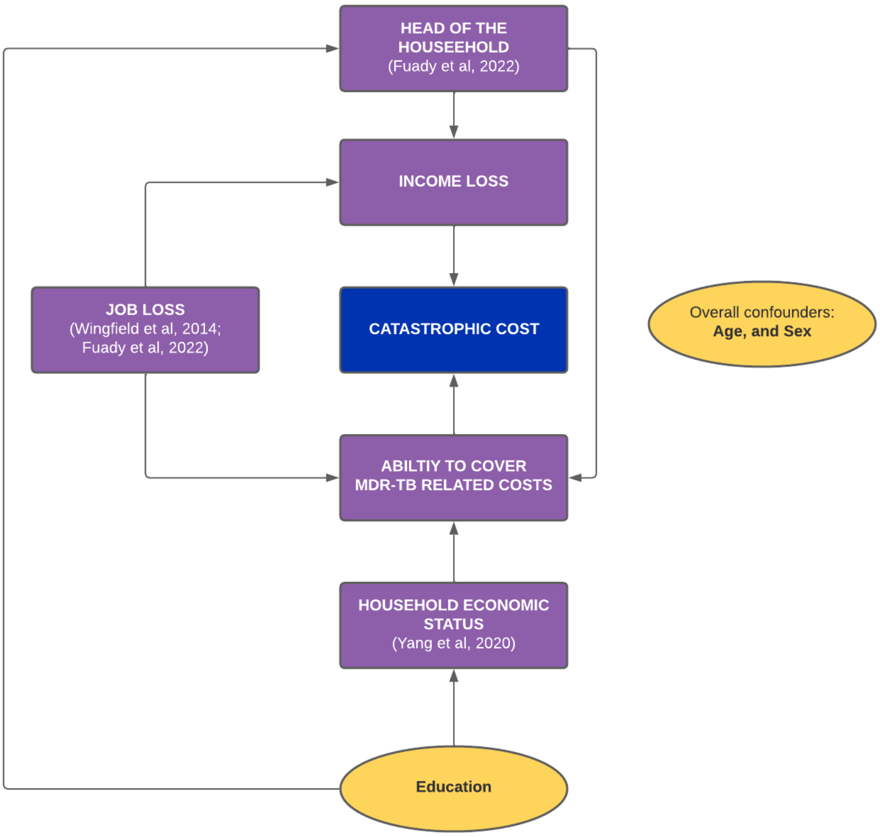

Figure S3 Conceptual Framework guiding the analysis of risk factors (purple) associated with the outcome of catastrophic costs (blue) and potential confounders (yellow).
