# Supplementary material for: Determinants of catastrophic costs among households affected by multi-drug resistant tuberculosis in Ho Chi Minh City, Viet Nam: a prospective cohort study
Source: BMC Public Health. 2023 Dec 3;23:2372. doi: 10.1186/s12889-023-17078-5 (PMC10693707; doi:10.1186/s12889-023-17078-5)
Supplement: Supplementary file 3 — Additional file 3. Patient cost survey for the first interview timepoint. [file 12889_2023_17078_MOESM3_ESM.pdf]

	Part I. Patient information (to be obtained before interview)		
#	Question	**Answer categories** *(circle appropriate number or fill answer on the answer line)*	***Action for interviewer***<br>*The questions in part 1 are not part of the interview and should be pre-filled before the interview*
1.	**Date of Interview**	____/____/____	(Day/month/year)
2.	**Name of Province**		
3.	**Name of District**		
4.	**Place of interview**		
5.	**Interviewer Name**		
6.	**Category of treating facility**	1. Commune Health Station<br>2. District TB Unit<br>3. Mobile chest X-ray event<br>4. Pharmacy/Drugstore<br>5. Private clinic<br>6. Public hospital/District Health Center - where the patient is registered<br>7. Public hospital/ District Health Center - **not** where the patient is registered<br>8. Private hospital<br>9. Herbalist/traditional practitioners<br>10. Other_____________________________	*The "treating facility" is the place where the patient's treatment card is kept*
7.	**Name of patient**		
8.	**Gender**	1. Male<br>2. Female	
9.	**Year of birth**	____ ____ ____ ____	
10.	**Study ID**	____ ____ ____ -____ ____ ____ -____ ____<br>(City code) (District code) (patient #)	*Generate study ID following this format ABC-XYZ-12, in which: ABC is the city code- (HN for Hanoi, HP for Hai Phong, HCM for HCMC), the district code is 2 or 3 letters and/or numbers. And 12 is the sequential number assigned for each patient within a district, ranging from 01 to 99.*<br><br>*For example the first patient from district Hong Bang in Hai Phong is: HP-HB-01. The 8th patient in District 8 in HCMC is: HCM-Q8-08. The 23rd patient from Hai Ba Trung in Hanoi will be: HN-HBT-23.*

Part II. Informed consent

Introduction to the patient:

My name is________________________. The organization I am working for, Friends for International TB Relief, is interested in the costs that people face when they are treated for TB as well as the costs faced while seeking health care before the diagnosis of TB.

The information that you choose to share will be used for research purposes. It will be shared with other researchers for further analysis and published, but all your personal information will first be deleted in order to ensure full confidentiality.

It is important for you to understand that your participation in this study is completely voluntary. We would be really grateful if you would agree to participate in this study, but do feel free to decline. If you decline, there will be no consequence for you and you will receive all the care and treatment you need at the health facility as usual. If you decline to participate you will not lose any benefit that you are entitled to such as receiving care and support that is provided at the clinic.

If you decide to participate, I would like to stress that you will not receive reimbursements from the study organisers for the expenses that you report on in this interview. However, your eligibility for existing reimbursement schemes will be unaffected.

If you choose to participate in this study, you may still withdraw from the study at any stage without giving any explanation for your withdrawal. Your answers will be kept confidential. At some point I will ask you about your personal income (revenue) and the income of your household. We will NOT provide this information to any tax or welfare authorities, even after the study has been completed.

In charge of this study is the Principal Investigator: (Luan Vo Nguyen Quang). The outcome of this study will be disseminated in an open source journal and you may request a copy from the principal investigator.

This survey will take approximately 60-90 minutes.

**#**	**Question**	**Answer categories *(circle appropriate number or fill answer on the answer line)***	***Action for interviewer***
	**Do you have any questions?**	*(No response recorded)*	*Answer patient's questions*
11	**Do you want to participate?**	1. Yes<br>2. No	*Yes → Thank you! Collect signature and go to interview*<br>*No → Ask Q11a*
11a	**-> Why not?**	1. Language not good enough<br>2. Time constraint<br>3. Not comfortable<br>4. Other, specify: _______________	*End the interview here having filled part I from patient card*<br>*This form should be signed by the child under 18 and guardian.*

Patient and/or Guardian Signature____

	**Inclusion or exclusion**

12	**Decision about inclusion or exclusion**	1. Included<br>2. Excluded	*Included → skip to Q13*<br>*Excluded-> Ask 12b*
12a	**->If excluded, reason for exclusion**	1. No informed consent<br>2. Treatment registration group is "other"	*After completing this question, the survey is completed for this patient excluded from the survey.*
13	**Interviewee identity**	1. Patient<br>2. Guardian<br>3. Other (please name) ______________________	

Part III- Costs before the current TB treatment (filled for new cases in intensive phase only)

	Out-of-pocket expenditure, reimbursements and time loss before and during TB diagnosis (before start of TB treatment)						
14	**Do you currently have any health insurance?**	1. Yes<br>2. No					*Yes → Ask Q14a*<br>*No → Skip to Q15*
14a	**-> What type of health insurance do you have?**	1. Mandatory- company purchases for employees<br>2. Voluntary- Paid out of pocket (typically for workers in the informal sector)<br>3. Private<br>4. Other:_______________________					*Ask if Q14 is 1. Yes*<br>*Multiple responses allowed.*
14b	**-> What is the health insurance number?**	______					*Ask if Q14 is 1. or 2.*<br>*Review health insurance card and collect type of insurance. Limit to 1-5.*
14c	**-> Select health insurance 2 letter code:**	BT<br>CA<br>CB<br>CC<br>CH<br>CK<br>CN<br>CS<br>CT<br>CY<br>DK	DN<br>DT<br>GB<br>GD<br>HC<br>HD<br>HG<br>HN<br>HS<br>HT<br>HX	KC<br>LS<br>MS<br>NN<br>NO<br>PV<br>QN<br>SV<br>TA<br>TB<br>TC	TE<br>TK<br>TN<br>TQ<br>TS<br>TY<br>XB<br>XD<br>XK<br>XN		*Ask if Q14 is 1. or 2.*<br>*Select one based on the insurance card. See interviewer guidelines at the end for more details.*

14d	**-> What percentage of health expenditure does your insurance cover?**	____%	*Ask if Q14 is 1. or 2.*<br>*See interviewer guidelines at the end for more details.*
14e.	**-> At which hospital are you registered?**	____	*Ask if Q14 is 1. or 2.*
15	**Did you experience symptoms of TB before you were diagnosed with this TB episode?**	1. Yes<br>2. No	*First construct a timeline of events, either starting with the first TB symptom, or start with time of TB diagnosis and work backwards. To help the patient remember when the illness started, you can ask which TB symptom was first experienced.*<br><br>*Probe for **cough, weight loss, chest pain, night sweats**.*<br><br>*If there is a problem defining the difference between TB symptoms and other health problems, ask which symptom led the patient to seek care, then ask when that symptom first occurred or became worse and started to worry the patient.*<br><br>*If answer to Q15 is 2. No, skip to Q16*
15a	**-> When did you first experience symptoms of TB for this TB episode?**	Number of weeks before treatment started: _________	

16	**Before your TB treatment started, did you seek care or advice for your symptoms of the current illness at either hospitals, commune health stations, district TB units, district health centres, pharmacies, or a private doctor?**	1. Yes*<br>2. No	*Yes → Fill out table for Q16a and Q16b*<br>*No → Skip to Q18 on page 7*<br><br>**Include the visits for diagnosis and enrollment in Q16 & Q17*
	**-> We would like to construct a timeline for every visit that you made to different providers for the symptoms of your current illness.** *(Enter in chronological order in 16a and 16b)*	**16a. Provider type**<br>1. Commune Health Station<br>2. District TB Unit<br>3. Mobile chest X-ray event<br>4. Pharmacy/Drugstore<br>5. Private clinic<br>6. Public hospital/District Health Center - where the patient is registered<br>7. Public hospital/ District Health Center - **not** where the patient is registered<br>8. Private hospital<br>9. Herbalist/traditional practitioners<br>10. Other___________________________	**16b. How many weeks before starting TB treatment in the current facility did you visit each of these providers?**
	**1st visit**	Provider category: ________	Number of weeks before treatment started: ________
	**2nd visit**	Provider category: ________	Number of weeks before treatment started: ________
	**3rd visit**	Provider category: ________	Number of weeks before treatment started: ________
	**4th visit**	Provider category: ________	Number of weeks before treatment started: ________
	**5th visit**	Provider category: ________	Number of weeks before treatment started: ________
	**6th visit**	Provider category: ________	Number of weeks before treatment started: ________
	**7th visit**	Provider category: ________	Number of weeks before treatment started: ________
	**8th visit**	Provider category: ________	Number of weeks before treatment started: ________
	**9th visit**	Provider category: ________	Number of weeks before treatment started: ________
	**10th visit**	Provider category: ________	Number of weeks before treatment started: ________
	**11st visit**	Provider category: ________	Number of weeks before treatment started: ________
	**12nd visit**	Provider category: ________	Number of weeks before treatment started: ________

17. How much money and time did you spend for each of these visits before you were diagnosed with TB, including the visit when you actually received your diagnosis? (For patient, guardian and the person accompanying the Patient)

				Medical out-of-pocket payments, (Total per visit) (A)									Non-medical out-of-pocket payments, (Total per visit) (B)				Health insurance contribution	
Visit *(corresponds with table for Q16)*	Provider Type (corresponds to Q16)	# of days hospitalized (for hospitalizations only)	Travel time and time spent for visit (Hours)	Daily room charges (for hospitalizations only) A1	Consultation fee (total for stay) A2	Radiography and other imaging A3	Lab tests A4	Other procedure A5	Medicines (do not include TB medicines) A6	TB medicines (do not include free meds) A7	Other medicines, incl nutritional supplements A8	Un-itemized medical payments A9	Travel B1	Food during health care visit or hospital stay B2	Other, including accommodation B3	Un-itemized Non-medical out-of-pocket payments B4	On this visit did you use health insurance?	Health insurance reimbursement to patient (C)
1st																	Y/N	
2nd																	Y/N	
3rd																	Y/N	
4th																	Y/N	
5th																	Y/N	
6th																	Y/N	
7th																	Y/N	
8th																	Y/N	
9th																	Y/N	
10th																	Y/N	
11st																	Y/N	
12nd																	Y/N	

	**Part IV. Cost during current TB/MDR-TB treatment (to be filled for all patients)** *This section refers to the patient's current treatment phase only*		
18	**After starting your TB treatment until now, did you seek care or advice for your symptoms of the current illness at either hospitals, commune health stations, District TB Units, District Health Centers, pharmacies, or a private doctor?**	1. Yes<br>2. No	*If yes, answer 18a and 18b; If no, skip to Q19.*<br>*This question only concerns medical visits that are not routine, not scheduled as part of the TB treatment. This question does not include DOT visits, drug pick-ups, outpatient visits for follow-up.*
	**-> Let's construct a timeline for every visit that you made to different providers for the symptoms of your current illness, since you began TB treatment.** *(Enter in chronological order in 18a and 18b)*	**18a. Provider type**<br>1. Commune Health Station<br>2. District TB Unit<br>3. Mobile chest X-ray event<br>4. Pharmacy/Drugstore<br>5. Private clinic<br>6. Public hospital/District Health Center - where the patient is registered<br>7. Public hospital/ District Health Center - **not** where the patient is registered<br>8. Private hospital<br>9. Herbalist/traditional practitioners<br>10. Other___________________________	**18b. Number of weeks**
	**1st visit**	Provider category: ________	Number of weeks since treatment started: ________
	**2nd visit**	Provider category: ________	Number of weeks since treatment started: ________
	**3rd visit**	Provider category: ________	Number of weeks since treatment started: ________
	**4th visit**	Provider category: ________	Number of weeks since treatment started: ________
	**5th visit**	Provider category: ________	Number of weeks since treatment started: ________
	**6th visit**	Provider category: ________	Number of weeks since treatment started: ________
	**7th visit**	Provider category: ________	Number of weeks since treatment started: ________
	**8th visit**	Provider category: ________	Number of weeks since treatment started: ________
	**9th visit**	Provider category: ________	Number of weeks since treatment started: ________
	**10th visit**	Provider category: ________	Number of weeks since treatment started: ________
	**11st visit**	Provider category: ________	Number of weeks since treatment started: ________
	**12nd visit**	Provider category: ________	Number of weeks since treatment started: ________

19.	**Are you currently hospitalized?**	1. Yes<br>2. No	*If yes, add this hospitalization to Q18a and 18b in chronological order*
20	**Have you been previously hospitalized or sought emergency care since you started TB treatment and because of TB?**	1. Yes<br>2. No	*If yes, add these hospitalizations/emergency care to Q18a and 18b in chronological order*<br>*Concerns only hospitalization/emergency care during the current treatment phase: Does not include hospitalizations/emergency care before the current TB treatment started. The hospitalizations/emergency care prior to TB treatment started should be filled in part III.*
21	**Did somebody in your household accompany you for your last hospitalization/unplanned visit?**	1. Yes<br>2. No	*If there were no outpatient visits/hospitalizations/emergency care in Q18, Q19 or Q20, then skip to Q23 on page10.*

22. About how much money and time did you spend for each of these hospitalizations/emergency care/unplanned outpatient visits? (For patient, guardian and/or the person accompanying the patient)

				Medical out-of-pocket payments, (Total per visit) (A)									Non-medical out-of-pocket payments, (Total per visit) (B)				Health insurance contribution	
Visit *(corresponds with table for Q18)*	Provider Type (corresponds to Q18)	# of days hospitalized (for hospitalizations only)	Travel time and time spent for visit (Hours)	Daily room charges (for hospitalizations only) A1	Consultation fee (total for stay) A2	Radiography and other imaging A3	Lab tests A4	Other procedure A5	Medicines (do not include TB medicines) A6	TB medicines (do not include free meds) A7	Other medicines, incl nutritional supplements A8	Un-itemized medical payments A9	Travel B1	Food during health care visit or hospital stay B2	Other, including accommo-dation B3	Un-itemized Non-medical out-of-pocket payments B4	On this visit did you use health insurance?	Health insurance reimbursement to patient (C)
1st																	Y/N	
2nd																	Y/N	
3rd																	Y/N	
4th																	Y/N	
5th																	Y/N	
6th																	Y/N	
7th																	Y/N	
8th																	Y/N	
9th																	Y/N	
10th																	Y/N	
11st																	Y/N	
12nd																	Y/N	

	Costs for DOT and food costs during ambulatory care *(DOT (Directly observed treatment) visit is for the supervision of daily intake of medicines, i.e, what is done every day. These questions are not referring to less frequent trips to pick up drugs (e.g., weekly), which are explored from Q24 onwards.)*		
23.	**On a daily basis, do you currently take your medicines yourself without supervision or support (self-administered) or do you have a treatment supervisor or supporter (DOT)?**	1. Self-administered<br>2. DOT via DTU<br>3. DOT via DTU/commune health station<br>4. DOT via private provider	• *If 1. self-administered skip to Q24*<br>• *If 2. DOT via DTU or 3. DTU/commune health station or 4.* DOT via private provider, *ask Q23a- Q23g*
23a.	**-> For any form of DOT, how many times a week?**	________ Times/week	*The maximum will be 7 times a week*
23b.	**-> Who is your DOT provider/supporter?**	1. Commune health station<br>2. DTU officer<br>3. Community health workers (CTV) or TB counsellors (TVV)<br>4. Work place<br>5. Family member<br>6. Doctor or other health care staff at the private provider<br>7. Other_________________	
23c.	**-> How long did the last DOT visit take, including travel time and waiting time (total turnaround time)?**	_________Minutes	
23d.	**-> What was the cost of transport (return) for the last DOT visit, including parking costs, in total for you and any accompanying household member?**	_________ VND	
23e.	**-> How much did you spend on food, drinks and accommodation for the last DOT visit (on the road, while waiting, lunch etc.), in total for you and any accompanying household member?**	_________ VND	
23f.	**-> If there were any other fees or costs, how much did you spend on other fees during your last DOT visit?**	_________ VND	*Include: plastic bags for sputum transport, cost of injections, travel for a DTU officer to the patient's home, etc.*<br>*If no other costs, then answer 0 VND*
23g.	**-> Did somebody in your household accompany you for your last DOT visit?**	1. Yes<br>2. No	

	Costs of picking up drugs and food costs during ambulatory care<br>*This section does not concern DOT visits, which should have recorded in the last section, but should filled if patient or other household member picks up drugs for either bringing to DOT provider or for self-administered treatment.*		
24.	**Do you or a household member pick up TB drugs (for self-administered treatment or to bring to your DOT supervisor/supporter)?**	1. Yes<br>2. No	*If patient is on DOT and patient or household member is **not** picking up drugs to bring to DOT provider, then the answer is no.*<br>*If yes, then answer 24a.-24g.*<br>*If no, skip to Q25 on page 12*
24a.	**-> How often do you or a household member pick up TB drugs in the current treatment phase?**	1. Every day<br>2. Every week<br>3. Every 2 weeks<br>4. Every month<br>5. Other_____________	
24b.	**->Where do you or your household member pick up your TB drugs?**	1. Commune health station<br>2. District TB unit<br>3. Other:_________	*If the patient has visited different places, tick the most recent one.*
24c.	**-> What accommodation cost did you and any accompanying household member have when you last picked up drugs?**	_________ VND	
24d.	**-> How long did the last visit to pick up drugs take, including travel time and waiting time (total turnaround time)?**	_________Minutes	
24e.	**-> What was the cost of transport (return) last time you picked up drugs, including parking costs, in total for you and any accompanying household member?**	_________ VND	
24f.	**-> How much did you spend on food and drinks last time you picked up drugs (on the road, while waiting, lunch etc.), in total for you and any accompanying household member?**	_________ VND	
24g.	**-> If there were any other fees or costs, how much did you spend on these other fees the last time you picked up drugs, in total, for you and any accompanying household member?**	_________ VND	*If no other costs, then answer 0 VND*
24h.	**-> Did somebody in your household accompany you for your last <u>visit to pick up drugs</u> <u>or did they pick up drugs for you</u>?**	1. Yes<br>2. No	

	Cost during outpatient visits for medical follow-up (see the doctor or nurse, have tests)		
25.	**How many TB-related medical follow-up visits have you had so far (to see the doctor or nurse, have follow- up tests, etc.)?**	_________ Times	*This concerns medical follow-up visits or follow-up tests that are scheduled by the doctor as part of the TB treatment. It does not include DOT visits or visits to pick up drugs or unplanned outpatient visits.*
26.	**How long did the last follow-up medical outpatient visit take, including travel time and waiting time (total turnaround time)?**	_________ Minutes	
27.	**What was the cost of transport (return) at the last follow-up medical outpatient visit, including parking, in total for you and any accompanying household member?**	_________ VND	*Cost related to the latest visit. If the interview takes place at the end of such a visit use the costs for the present visit for Q27-36*
28.	**How much did you spend on food and drinks at the last follow-up medical outpatient visit (on the road, while waiting, lunch etc.), in total for you and any accompanying household member?**	_________ VND	
29.	**What accommodation cost did you have for the last follow-up medical outpatient visit, in total, for you and any accompanying household member?**	_________ VND	
30.	**What fees did you pay during your last follow-up medical outpatient visit for <u>registration/consultation</u>?**	_________ VND	
31.	**What fees did you pay during your last follow-up medical outpatient visit for <u>radiography and other imaging</u>?**	_________ VND	
32.	**What fees did you pay during your last follow-up medical outpatient visit <u>for tests, TB tests and others</u>?**	_________ VND	
33.	**What fees did you pay during your last follow-up medical outpatient visit for <u>other procedures</u>?**	_________ VND	
34.	**What fees did you pay at your last follow-up medical outpatient visit for <u>TB medicines</u>, including prescriptions for medicines bought outside the facility?**	_________ VND	
35.	**What fees did you pay during your last follow-up medical outpatient visit for <u>other medicines</u>, including nutritional supplements?**	_________ VND	
36.	**What <u>other fees</u> not listed in the previous questions did you pay during your last follow-up medical outpatient visit?**	_________ VND	*Examples include: masks, health staff fee (unofficial)*
37.	**Did somebody in your household accompany you for your <u>last medical follow-up visit</u>?**	1. Yes<br>2. No	
	Costs for nutritional/food supplements		
38.	**Do you buy any nutritional supplements <u>outside your regular diet</u> because of the TB illness, for example vitamins, meat, energy drinks, or fruits as recommended by health care staff?**	1. Yes<br>2. No	*If yes, ask Q38a*<br>*If no, skip to Q39*
38a.	**-> How much did you spend on nutritional supplements (vitamins, meat, energy drinks, or fruits) in the past week approximately?**	_________ VND	Record here the actual costs for supplements in the past week.

	Costs for relocation related to TB treatment		
39	**During this TB episode, did your (the patient and his/her household) housing situation change because of your TB diagnosis/treatment?**	1. Yes<br>2. No	If yes, ask Q39a,b,c
39a.	**Do you now spend more/less per month for the new accommodation? (including rent, utilities)**	1. More<br>2. Less<br>3. Same	
39b.	**-> How much is the difference per month?**	_________ VND	Put in 0 if answer to 39a = 3 Same
39c.	**-> How much were the one-time costs in total related to moving to the new accommodation?**	_________ VND	E.g. transport of goods
	Time loss for guardians<br>• ***Note: out-of-pocket costs of transport, food, accommodation for guardian should be included in questions on Part IV (tables).***		
40.	Earlier in the survey, you indicated that others have accompanied you as you sought care for TB and we collected the out-of-pocket costs for the person who accompanied you. **Can you identify the last time someone accompanied you as you sought healthcare and the type of visit that you were going for?**	0. No one has accompanied me<br>1. Hospitalization/emergency care/unplanned outpatient visits<br>2. DOT visit<br>3. Drug pick-up<br>4. Medical follow-up visit	*If yes, to Q21, Q23g, Q24h or Q37, then responses to Q39 should be 1-4.*<br>*If Q40 is 0. No one accompanied me, then skip to Q41*
40a.	**->Who accompanied you to that visit?**	1. Grandfather<br>2. Grandmother<br>3. Father<br>4. Mother<br>5. Uncle<br>6. Aunt<br>7. Husband<br>8. Wife<br>9. Brother<br>10. Sister<br>11. Cousin (male)<br>12. Cousin (female)<br>13. Child (son)<br>14. Child (daughter)<br>15. Friend<br>16. Other household member_________	
40b.	**->During that last visit, did the person who accompanied you, take time off work?**	1. Yes<br>2. No	
40c.	**->Did that person lose any income as they accompanied you for the visit?**	1. Yes<br>2. No	*If Q39c is 1. Yes, then ask 39d.*<br>*If Q39c is 2. No, then skip to Q40*
40d.	**->How much income would you estimate that the person who accompanied you lost during your last visit?**	_________ VND	

	**PART V: Social position**		
41	**How many people are in your household?**	______ people	*The interviewer should work with the interviewee to list out the names of the people who live under the same roof as the patient, share a kitchen and have been living there for at least one month. See template in interviewer guidelines.*
41a	**-> Number of adults**	_________ Adults	
41b	**-> Number of children**	_________ Children	
42.	**Are you the head of your household?**	1. Yes<br>2. No	*The HoH is the person listed in the family book as the HoH -or- the person that family members trust and respect most.*
42a.	**-> Who is the head of your household?**	1. Grandfather<br>2. Grandmother<br>3. Father<br>4. Mother<br>5. Uncle<br>6. Aunt<br>7. Husband<br>8. Wife<br>9. Brother<br>10. Sister<br>11. Cousin (male)<br>12. Cousin (female)<br>13. Child (son)<br>14. Child (daughter)<br>15. Friend<br>16. Other household member_________	*Ask if 42 is 2.No*<br>*Skip if 42 is 1.Yes*
42b.	**->What education level did the head of the household complete?**	*1. Not literate*<br>*2. Literate*<br>*3. Primary school*<br>*4. Secondary school*<br>*5. High school*<br>*6. Technical school*<br>*7. College/University*<br>*8. Post-graduate*	*Ask if 42 is 2.No*<br>*Skip if 42 is 1.Yes*
42c.	**->How many total years of education did the head of household complete?**	_________ *Years*	*Ask if 42 is 2.No*<br>*Skip if 42 is 1.Yes*

43.	**What education level did you complete?**	*1. Not literate*<br>*2. Literate*<br>*3. Primary school*<br>*4. Secondary school*<br>*5. High school*<br>*6. Technical school*<br>*7. College/University*<br>*8. Post-graduate*	*If patient is under 15 years, this question is for the guardian.*
43a.	**How many total years of education did you complete?**	________ *Years*	
44.	**What is your main occupation?**	1. School student<br>2. Technician<br>3. Service<br>4. Factory worker<br>5. Farmer<br>6. Government employee<br>7. Teacher<br>8. Retiree<br>9. Homemaker<br>10. Unemployed<br>11. Manual labor<br>12. Microenterprise owner<br>13. Other (specify): ________	*If patient is under 15 years, this question is for the guardian. This section will be adapted to the local occupation definitions (ref. Statistical dpt/Household survey categories)*
45.	**What was your primary employment, or normal work, or normal other main activity before you contracted TB?**	0. Unemployed<br>1. Formal paid work<br>2. Informal paid work<br>3. Retired<br>4. Student<br>5. Housework<br>6. Other (specify): ________	*If patient is under 15 years, this question is for the guardian. This refers to the time before TB symptoms developed. Name all options first*<br><br>*"3.Retired" is defined as when a person is no longer working because of their age. It does not only refer to government employees who receive a pension.*
46.	**What is your primary employment, or normal work, or normal other main activity now?**	0. Unemployed<br>1. Formal paid work<br>2. Informal paid work<br>3. Retired<br>4. Student<br>5. Housework<br>6. Other (specify): ________	*If patient is under 15 years, this question is for the guardian. This refers to the time after TB symptoms developed. Name all options first*

	Constructing a socio-economic status index with household asset questions.		
47.	**What is your usual main source of <u>drinking</u>/ cooking water?**	1. Piped or Bottled<br>2. Well<br>3. Other	*Other includes all sources that are not from a piped source, bottle, or well. This includes natural spring, borehole, rainwater, etc.*
48.	**What kind of toilet facilities do you have?**	1. Flush toilet<br>2. Other	
49.	**Does your household have?**		
	Electricity	1. Yes 2. No	
	Mobile Telephone	1. Yes 2. No	
	Fixed Telephone	1. Yes 2. No	
	Refrigerator	1. Yes 2. No	
	Computer	1. Yes 2. No	
	Fan	1. Yes 2. No	
	Watch	1. Yes 2. No	
	Bicycle	1. Yes 2. No	
	Television	1. Yes 2. No	
	Manual scooter	1. Yes 2. No	
	Automatic scooter	1. Yes 2. No	
	Car	1. Yes 2. No	
	Sewing machine	1. Yes 2. No	
	Stereo equipment	1. Yes 2. No	
	Air conditioner	1. Yes 2. No	
	Washing machine	1. Yes 2. No	
	Drying machine (dryer)	1. Yes 2. No	
	Water heater	1. Yes 2. No	
	Gas stove	1. Yes 2. No	
	Induction cooker	1. Yes 2. No	
	Rice cooker	1. Yes 2. No	
	Vacuum cleaner	1. Yes 2. No	
	Dehumidifier	1. Yes 2. No	
		1. Yes 2. No	
	Microwave	1. Yes 2. No	
	Oven	1. Yes 2. No	
	Fax machine	1. Yes 2. No	
	Wheelchair	1. Yes 2. No	

	***Income (reported) before contracting TB** (This refers to the time before TB symptoms developed.)*		
50.	**Were you the person who earned the highest income in your household before you contracted TB?**	1. Yes<br>2. No	*For this section, if patient is under 15 years, this question is for the guardian.*
51.	**How many hours a week were you working before you contracted TB?**	_________ hours/week	
52.	**If you were in paid work, how much do you estimate your take-home (net) income from labour related activities, per month was before you contracted TB?**	_________ VND	*If patient is under 15 years, this question is for the guardian. If the patient reports 0 net income, then the interviewer should record 0 in Q52. and at the end of the survey, in Q65, details about how the person managed to live without income should be recorded.*
53.	**How much was your estimated income each month from other sources (not labor-related) before you contracted TB?**	_________ VND	*Include income from owning rentals, interest from investments, etc… If no other income, mark 0 VND.*
54.	We are interested in understanding the income of your household before you contracted TB. We previously listed all of the people in your household. **Can you estimate how much income each person in your household earned (take-home/net income) per month from labour related activities before you contracted TB?**<br><br>*Enter:* Estimated net income from labour related activities of the household was per month, before you contracted TB?	 <br><br><br><br><br>_________ VND	*The interviewer should go through the list of people created in Q40. and ask if each person was working before the patient contracted TB. The interviewer should write down the estimated income from labour related activities for each person. Once the interviewer has obtained an estimated income for each household member, the interviewer should add the estimated income for each person in the household and enter the total here.*
55.	**Can you estimate how much income each person in your household earned (take-home/net income) per month from non-labour related activities before you contracted TB?**	_________ VND	*Include income from owning rentals, interest from investments, etc… If no other income, mark 0 VND.*

	Income changes and social consequences		
56.	**Are you currently working?**	1. Yes<br>2. No	*For this section, if patient is under 15 years, this question is for the guardian.*
56a	**-> If yes, how many hours per week are you working now?**	_________ hours/week	*Ask if Q56 is 1.Yes*
56b.	**-> If yes, what is your current monthly take-home income (net)?**	__________ VND	*Ask if Q56 is 1.Yes*
56c.	**-> If yes, how many working days have you lost because of TB?**	_________ Days	*Ask if Q56 is 1.Yes*<br>*Working days: e.g., if a patient was not able to work for 5 half days and lost income for these, the number of days lost is 0.5*5=2.5.*
56d.	**-> If no, when did you stop working?**	_____/_____/_____	*Ask if Q56 is 2. No*<br>*Day/month/year*
56e.	**-> If no, why did you stop working?**	1.Illness related to TB<br>2. Not related to TB	*Ask if Q56 is 2. No*
56f.	**-> If no, how many working days did you lose before you stopped working entirely?**	_________ Days	*Ask if Q56 is 2. No*<br>*Working days: e.g., if a patient was not able to work for 5 half days and lost income for these, the number of days lost is 0.5*5=2.5. Report for all days before job loss.*
57.	We are interested in understanding the current income of your household. We previously listed all of the people in your household. **Can you estimate how much income each person in your household currently earns (take-home income or net) per month from labour related activities?**<br><br>*Enter:* Estimated current net income from labour related activities of the household per month:	__________ VND	*The interviewer should go through the list of people created in Q40, which has the estimated income for each person from Q54. The interviewer should ask if each person on the list is currently working. The interviewer should write down the estimated income from labour related activities for each person. Once the interviewer has obtained an estimated current income for each household member, the interviewer should add the estimated income for each person in the household and enter the total here.*
58	**Can you estimate how much income each person in your household currently earns (take-home/net income) per month from non-labour related activities?**	_________ VND	*Include income from owning rentals, interest from investments, etc… If no other income, mark 0 VND.*

59.	**Did you or your household receive any social welfare payment or cash transfer after you were diagnosed with TB?**	0. No<br>1. Paid sick leave<br>2. Disability grant<br>3. Cash transfer for poor families<br>4. Cash transfer for other preferential policies<br>5. Other cash transfer	*Multi-select*<br>*If answer 0. No, skip to Q60*<br>*Answer 2,3,4 refers to the frequent cash transfers provided by governmental agencies/through governmental policies.*<br>*Answer 5 refers to the cash transfers provided by other types of organizations or individuals, including IRD/FIT/CHI social support program, charity programs, cash from relatives or friends who are not members of the household.*
59a.	**-> How much did you receive for paid sick leave in the last month?**	________ VND	*Ask if Q59=1*
59b.	**-> How much did you receive for the disability grant in the last month?**	________ VND	*Ask if Q59=2*
59c.	**-> Did program officers from the social support program assist you in obtaining a disability grant?**	1. Yes<br>2. No	*Ask if Q59=2*<br>*Only applicable if support was provided by IRD VN/FIT/CHI*
59d.	**-> If yes, how much support did the program officers help you receive from the disability grant in the last month?**	________ VND	*Ask if Q59c=1. Yes*<br>*Only applicable if support was provided by IRD VN/FIT/CHI*
59e.	**-> How much did you receive for cash transfers for poor families in the last month?**	________ VND	*Ask if Q59=3*
59f.	**-> Did the program officers from the social support program assist you in obtaining any cash transfers for poor families?**	1. Yes<br>2. No	*Ask if Q59=3*<br>*Only applicable if support was provided by IRD VN/FIT/CHI*
59g.	**-> If yes, how much support did the program officers help you receive from the cash transfers for poor families in the last month?**	________ VND	*Ask if Q59f=1. Yes*<br>*Only applicable if support was provided by IRD VN/FIT/CHI*
59h.	**-> How much did you receive for cash transfers for other preferential policies in the last month?**	________ VND	*Ask if Q59=4 Include payments to elderly people and any other cash transfers from other preferential policies*
59i.	**-> Did the program officers from the social support program assist you in obtaining any other cash transfers?**	1. Yes<br>2. No	*Ask if Q59=4*<br>*Only applicable if support was provided by IRD VN/FIT/CHI*
59j.	**-> If yes, how much support did the program officers help you receive from other cash transfers in the last month?**	________ VND	*Ask if Q59i=1. Yes*<br>*Only applicable if support was provided by IRD VN/FIT/CHI*
59k.	**How much did you receive for other routine cash transfers in the last month?**	________ VND	*Ask if Q59=5*
59l.	**How much did you receive in total from one-time and infrequent cash transfers after you were diagnosed with TB?**	________ VND	*Ask if Q59=5.*<br>*All one-time and infrequent cash transfers should be recorded here. Do not include any payments which have been recorded in 59a-k.*

60.	**Do you currently receive vouchers or goods in kind to cope with TB illness?**	1. Yes<br>2. No	*If no, skip to Q61.*
60a.	**-> From whom do you receive the voucher/ goods**	1. Government<br>2. NGO<br>3. Employer<br>4. Private donation<br>5. Other: _________	*Ask if Q60 is 1.Yes*<br>*Multi-select*<br>*Vouchers and goods provided by IRD/FIT/CHI will be counted as NGO.*
60b.	**-> How much do you receive in travel vouchers in the last month?**	_________ VND	*Ask if Q60 is 1.Yes*
60c.	**-> Did the program officers from the social support program assist you in obtaining these travel vouchers?**	1. Yes<br>2. No	*Ask if Q60b >0*<br>*Only applicable if support was provided by IRD VN/FIT/CHI*
60d.	**-> If yes, how much support did the program officers help you receive from travel vouchers in the last month?**	_________ VND	*Ask if Q60c. is 1.Yes*
60e.	**-> How much do you receive in food support in the last month?**	_________ VND	*Ask if Q60 is 1.Yes*
60f.	**-> Did the program officers from the social support program assist you in obtaining this food support?**	1. Yes<br>2. No	*Ask if Q60e >0*<br>*Only applicable if support was provided by IRD VN/FIT/CHI*
60g.	**-> If yes, how much support did the program officers help you receive the food support in the last month?**	_________ VND	*Ask if Q60f. is 1.Yes*
60h.	**-> How much do you receive in other enablers in the last month?**	_________ VND	*Ask if Q60 is 1.Yes*
60i.	**-> Did the program officers from the social support program assist you in obtaining these other enablers?**	1. Yes<br>2. No	*Ask if Q60h >0*<br>*Only applicable if support was provided by IRD VN/FIT/CHI*
60j.	**-> If yes, how much did the program officers help you receive the other enablers in the last month?**	_________ VND	*Ask if Q60i. is 1.Yes*
60k.	**How much did you receive from one-time and infrequent vouchers and in-kind support after you were diagnosed with TB?**	________ VND	*Ask if Q60 is 1.Yes*<br>*All one-time and infrequent vouchers and in-kind support should be recorded here, including gifts from relatives and friends who are not members of the household, charity, association. Do not include any in-kind transfers which have been recorded in 60b-j.*
61.	**How many rooms are there in the house, excluding the bathroom?**	________ Rooms	*All spaces in the house are considered rooms, even if used for storage or parking a motorbike. One space = 1 room. Exclude bathrooms.*
62.	**Besides yourself, does anyone else of your household receive treatment for TB?**	1. Yes<br>2. No	
62a.	**>How many people in your household receive treatment for DSTB?**	________ people	Ask if Q62 is 1.Yes
62b.	**>How many people in your household receive treatment for DRTB?**	________ people	Ask if Q62 is 1.Yes
62c.	**>How many people in your household receive treatment for LTBI?**	________ people	Ask if Q62 is 1.Yes

63.	**Has the TB illness affected your social or private life in any way?**	1. No<br>2. Food insecurity<br>3. Divorce or Separated from spouse/partner<br>4. Loss of Job<br>5. Interrupted schooling<br>6. Social exclusion<br>7. Isolation from family<br>8. Experienced stigma<br>9. Self-limiting contact/communication with others<br>10. Other_________	More than one category allowed.
	Coping		
64	**Did you borrow or receive any money to cover costs incurred since you started TB treatment?**	1. Yes<br>2. No	*Pawning items should be included here since it is a loan secured by an asset. Do not include pre-treatment loans.*
64a.	**-> If yes, how much did you borrow (in total) or receive?**	_________ VND	*Ask If Q64 is 1.Yes*
64b.	**-> From whom did you borrow?**	1. Family<br>2. Neighbors/friends<br>3. Private bank<br>4. Cooperative<br>5. Employer<br>6. "Unofficial lender" (Black market)<br>7. Pawnshop<br>8. Other: _________	*Ask If Q64 is 1.Yes. Multiple responses allowed.*<br>*If the patient took out a loan at a state-owned commercial bank, including*<br>- *Vietnam Bank for Agriculture and Rural Development - Agribank,*<br>- *Global Petro Sole - GP Bank,*<br>- *Ocean Bank,*<br>- *Construction Bank,*<br>*choose 8. Other and specify the bank*
64c.	**-> Have you started paying back the loan(s)?**	1. Yes<br>2. No	*Ask If Q64 is 1.Yes*
64d.	**-> How much of the principle have you paid back?**	_________ VND	*Ask If Q64c is 1.Yes*
64e.	**-> How much of the interest have you paid back?**	_________ VND	*Ask If Q64c is 1.Yes*
65.	**Have you sold any of your property to finance the costs incurred during TB treatment?**	1. Yes<br>2. No	
65a.	**-> If yes, what did you sell?**	1. Land<br>2. Livestock<br>3. Transport/vehicle<br>4. Household item<br>5. Farm produce<br>6. Gold/jewelry<br>7. Other :_________	*Ask if Q65 is 1.Yes*<br>*Multiple responses allowed. Circle all that are mentioned*

65b.	**-> How much money did you receive from the sale of all items of your property (in total)?**	_________ VND	*Ask if Q65 is 1.Yes*
65c.	**The assets that you sold, were they previously supporting the family income (or expenditure)?**	1. Yes<br>2. No	*Ask if Q65 is 1.Yes*
65d.	**-> If yes indicate monthly income previously generated by the assets**	_________ VND	*Ask if Q65c is 1.Yes*
65e.	**What is the estimated market value of all the property you sold?**	_________ VND	*Ask if Q65 is 1.Yes.*<br>*Market value refers to the value that could have been realized if the seller was not under pressure to sell quickly to raise funds.*
66.	**Thank you for your cooperation! Is there anything you would like to ask or say?**		
**67.**	**Comments by Interviewer:**		

**Date** *(dd/mm/yyyy)*:	____/____/____	**Signature interviewer:**	____

Interviewer Guidelines

(To be printed and brought to every interview)

Fill out checklist before each interview:

Checklist for which parts of the questionnaire to fill for different treatment categories			
**Treatment category and treatment phase at time of interview**	**Questionnaire part III (tick when filled)**	**Questionnaire part IV (tick when filled)**	**Supervisor check**
**Not MDR**			
First line, new case, interviewed in the intensive treatment phase	*Filled* □	*Filled* □	
First line, new case, interviewed in the continuation treatment phase	*Do not fill*	*Filled* □	
**MDR**			
MDR, new case, interviewed in the intensive treatment phase	*Filled* □	*Filled* □	
MDR, new case, interviewed in the continuation treatment phase	*Do not fill*	*Filled* □	
MDR, relapse or re-treatment	*Do not fill*	*Filled* □	

General guidelines:

1. A duplicate of this signed questionnaire should be offered to the patient.
2. Questions should be read exactly as they appear in the column labeled Question.
3. If the interviewee responds to a question with a range, then the average (mean) of the range should be taken and recorded on the survey. For example, if you ask how for many weeks? And the patient responds with 2-3 weeks. Then the response 2.5 weeks should be entered on the form.

Health insurance guidelines for Question 14:

**Number 1**	SHI covers 100% (without limitation on percentage of reimbursement for some specific/special medicines, chemicals medical supplies and technological services) and transportation to the referral hospitals.	This SHI card goes along with first 2- letter abbreviations **CC, TE.**
**Number 2**	SHI covers 100% (with limitation on percentage of reimbursement for some specific/special medicines, chemicals medical supplies and technological services) and transportation to the referral hospitals.	This SHI card goes along with first 2- letter abbreviations **CK, CB, KC, HN, DT, DK, XD, BT, TS.**
**Number 3**	SHI covers 95% (with limitation on percentage of reimbursement for some specific/special medicines, chemicals medical supplies and technological services). SHI covers 100% of examination and treatment costs at communal hospitals and cost for 1 visit less than 15% of government base salary.	This SHI card goes along with first 2- letter abbreviations **HT, TC, CN**
**Number 4**	SHI covers 80% (with limitation on percentage of reimbursement for some specific/special medicines, chemicals medical supplies and technological services). SHI covers 100% of examination and treatment costs at communal hospitals and cost for 1 visit less than 15% of government base salary.	This SHI card goes along with first 2- letter abbreviations **DN, HX, CH, NN, TK, HC, XK, TB, NO, CT, XB, TN, CS, XN, MS, HD, TQ, TA, TY, HG, LS, PV, HS, SV, GB, GD.**
**Number 5**	SHI covers 100% including costs of examination and treatment outside the scope of reimbursement; costs of transportation.	This SHI card goes along with first 2-letter abbreviations **QN, CA, CY.**

Where to find the health insurance number and type on the health insurance card:

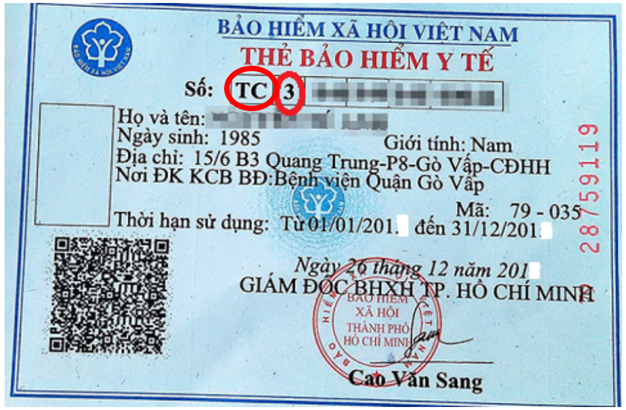

Instructions for Q17- Pre-treatment costs and Q22 Costs of hospitalizations and emergency care

- Fill one line per visit in chronological order. The visits should correspond with Q16 exactly.
- For all that don't apply, mark NA
- If there were payments for an item, but the patient cannot remember the amount, mark NR
- Add more rows if there were more visits than rows.

**Visits**	Includes outpatient visits as well as hospitalizations. Should be filled in chronological order, 1st visit=visit 1.
**Provider type**	Must correspond to Questions 16 and 21. The provider type is duplicated to ensure that Q16-Q17 and Q21-Q22 matches.
**Number of days hospitalized**	To be filled for each hospitalization. Should be filled in chronological order. If the patient was not hospitalized on this visit, then mark NA.
**Travel time and time spent for visit**	Hours spent to travelling to and from facility. This column measures the total time from leaving home to returning home, including the time waiting for outpatient visits. For hospitalizations, multiply the number of days by 24 hours and include hours.-
**A1- Daily room charges**	Fees for time spent in the hospital. Only for hospitalizations, and only to be filled if not covered by the other cost items (consultation fee, radiography etc.). If the patient was not hospitalized, then fill with NR.
**A2- Consultation fee**	Other charges paid by the patient, not covered under day charge, including direct payment to health care staff. For hospitalizations, include total out-of-pocket payments for the entire stay.
**A3- Radiography and other imaging**	Out-of-pocket payments for imaging investigation (X-rays, CT-scan, ultrasound), TB-specific and other.
**A4- Lab test fees**	Out-of-pocket payments for all tests, TB specific and others.
**A5- Other procedures**	Out-of-pocket payments for biopsy, bronchial lavage etc… but not surgery unrelated to TB.
**A6 - Medicines (do not include TB medicines)**	Any medicine that is not TB medicine
**A7 - TB medicines**	Any TB medicine (do not include the TB medicines that are provided free-of-charge)
**A8 - Other, including nutritional supplements**	Any other treatments paid out of pocket, such as nutritional supplements medically indicated. This is for all known costs that do not fit within A1-A7.
**A9 - Un-itemized costs**	If the patient did not receive a bill or does not know the cost breakdown for medical out-of-pocket payments, then leave A1-A8 blank and fill in column A9 for the estimate of all un-itemized costs.
**B1- Travel**	Out-of-pocket payments for travel to the facility, for both patient and any household member who accompanied the patient.
**B2- Food**	Out-of-pocket payments for additional food bought in relation to travelling the health care visit, and during visit or hospitalization, for both patient and any household member.
**B3- Other, including accommodation**	Includes out-of-pocket payments related to renting a room/bed during health care visits, and any other non-medical payments related to health care visit, for both patient and any household member.
**B4- Un-itemized costs**	If the patient cannot distinguish between the costs for categories B1-B3, but can estimate the total, then leave B1-B3 blank and fill out B4.
**On this visit did you use health insurance?**	Circle Yes, if the patient has health insurance (Q14) and the health insurance and submitted the health insurance information to the facility at the beginning of the visit. Circle No, if the patient does not have health insurance (Q14) or has health insurance and did not submit the information to the facility. Also circle No if the patient was at a medical facility where their insurance was not valid.
**Reimbursement from health insurance**	Amount reimbursed to patient through medical insurance (private or social security) so far, does not include expected future reimbursement. This is not the amount the health insurance pays on the patient's behalf- it is the money that the health insurance company returns to the patient.

Template for establishing household size and income in Questions 54, 55, 57 and 58.

Definition of a household: *People who live under the same roof as the patient, share a kitchen and have been living there for at least one month.*
Include the patient as a member of the household.

Number	Person identifier	Under 15 (X)	Over 15(X)	Question 54- estimated monthly net income from labor related activities before TB *(for total add all amounts in this column)*	Question 55- estimated monthly net income from non-labor related activities before TB *(for total add all amounts in this column)*	Question 57 estimated monthly net income from labor related activities after TB *(for total add all amounts in this column)*	Question 58- estimated monthly net income from non-labor related activities after TB *(for total add all amounts in this column)*
1							
2							
3							
4							
5							
6							
7							
8							
9							
10							
	**Total**						
