# Supplementary material for: Determinants of catastrophic costs among households affected by multi-drug resistant tuberculosis in Ho Chi Minh City, Viet Nam: a prospective cohort study
Source: BMC Public Health. 2023 Dec 3;23:2372. doi: 10.1186/s12889-023-17078-5 (PMC10693707; doi:10.1186/s12889-023-17078-5)
Supplement: Supplementary file 4 — Additional file 4. Patient cost survey for the second and third interview timepoint. [file 12889_2023_17078_MOESM4_ESM.pdf]

	Part I. Patient information (to be filled out before interview)		
**#**	**Question**	**Answer categories** *(circle appropriate number or fill answer on the answer line)*	***Action for interviewer***<br>*The questions in part 1 are not part of the interview and should be pre-filled before the interview*
0a	**Date of Interview**	____/____/____	(Day/month/year)
0b	**Interviewer Name**		
0c	**Name of patient**		
0d	**Study ID**	____ ____ ____ -____ ____ ____ -____ ____<br>(City code) (District code) (patient #)	*Generate study ID following this format ABC-XYZ-12, in which: ABC is the city code- (HN for Hanoi, HP for Hai Phong, HCM for HCMC), the district code is 2 or 3 letters and/or numbers. And 12 is the sequential number assigned for each patient within a district, ranging from 01 to 99.*<br><br>*For example, the first patient from district Hong Bang in Hai Phong is: HP-HB-01. The 8th patient in District 8 in HCMC is: HCM-Q8-08. The 23rd patient from Hai Ba Trung in Hanoi will be: HN-HBT-23.*
0e	**Interview number**	1. Interview #2<br>2. Interview #3<br>3. Interview #4	

Part II- Costs during the current phase of TB treatment			
1	**Since the last interview, did you seek care or advice for your symptoms of the current illness at either hospitals, commune health stations, district TB units, district health centers, pharmacies, or a private doctor?**	1. Yes<br>2. No	*Yes → Fill out table for Q1a and Q1b*<br>*No → Skip to Q2*<br>*This question only concerns medical visits that are not routine, not scheduled as part of the TB treatment. This question does not include DOT visits, drug pick-ups, outpatient visits for follow-up.*
	**-> We would like to construct a timeline for every visit that you made to different providers for the symptoms of your current illness.** *(Enter in chronological order in 1a and 1b)*	**1a. Provider type**<br>1. Commune Health Station<br>2. District TB Unit<br>3. Mobile chest X-ray event<br>4. Pharmacy/Drugstore<br>5. Private clinic<br>6. Public hospital/District Health Center - where the patient is registered<br>7. Public hospital/ District Health Center - **not** where the patient is registered<br>8. Private hospital<br>9. Herbalist/traditional practitioners<br>10. Other___________________________	**1b. How many weeks since the last interview did you visit each of these providers?**
	**1st visit**	Provider category: ________	Number of weeks since last interview: ________
	**2nd visit**	Provider category: ________	Number of weeks since last interview: ________
	**3rd visit**	Provider category: ________	Number of weeks since last interview: ________
	**4th visit**	Provider category: ________	Number of weeks since last interview: ________
	**5th visit**	Provider category: ________	Number of weeks since last interview: ________
	**6th visit**	Provider category: ________	Number of weeks since last interview: ________
	**7th visit**	Provider category: ________	Number of weeks since last interview: ________
	**8th visit**	Provider category: ________	Number of weeks since last interview: ________
	**9th visit**	Provider category: ________	Number of weeks since last interview: ________
	**10th visit**	Provider category: ________	Number of weeks since last interview: ________
	**11st visit**	Provider category: ________	Number of weeks since last interview: ________
	**12nd visit**	Provider category: ________	Number of weeks since last interview: ________

2.	**Are you currently hospitalized?**	1. Yes<br>2. No	*If yes, add this hospitalization to Q1a and 1b in chronological order*
3.	**Since the last interview, have you been hospitalized or sought emergency care because of TB?**	1. Yes<br>2. No	*If yes, add these hospitalizations/emergency care to Q1a and 1b in chronological order*<br>*If answers to Q1-3 are all no, then skip to Q4 on page 5*

Instructions for the table in Q3a.

- See table below, and ask for each item. Fill one line per visit.
- The table should be filled in chronological order.
- For all that don't apply, mark/select NA
- If there were payments for an item, but the patient cannot remember the amount, mark NR

Explanation of table headings:

- **Type of hospital**: fill in provider type according to categories in Q1a
- **Number of days hospitalized**: Include the current day if Q2 is Yes. Must be filled out for each hospitalization. Should be filled in chronological order.
- **Travel time and time spent for visit:** Hours spent travelling to and from facility. This column measures the total time from leaving home to returning home, including the time waiting for outpatient visits. For hospitalizations, multiply the number of days by 24 hours and include hours.
- **A1- Daily room charges:** Fees for time spent in the hospital. Only for hospitalizations, and only to be filled if not covered by the other cost items (consultation fee, radiography etc.). If the patient was not hospitalized, then fill with NR
- **A2- Consultation fee:** Other charges paid by the patient, not covered under day charge, including direct payment to health care staff. For hospitalizations, include total out-of-pocket payments for the entire stay
- **A3- Radiography and other imaging:** Any imaging investigation (x-rays, CT-scan, ultrasound), TB-specific and other
- **A4- Lab test fees**: includes all tests, TB specific and others, including cost of transporting samples, if paid by patient
- **A5- Other procedures:** includes biopsy, bronchial lavage, etc. but not surgery unrelated to TB
- **A6- Medicines (do not include TB medicines):** The total cost of medicines that are not TB medications.
- **A7- TB medicines:** fees for TB medicines only, bought inside or outside hospital. Do not include the value of free medicines.
- **A8- Other medicines, including nutritional supplements**: any other medicine, including nutritional supplements. This is for all known costs that do not fit within A1-A7.
- **A9- Un-itemized medical payments- I**f the patient did not receive a bill or does not know the cost breakdown for medical out-of-pocket payments, then leave A1-A8 blank and fill in column A9 for the estimate of all un-itemized costs.
- **B1- Travel:** out-of-pocket payment for travel to the facility (does not include income loss), for both patient and any household member who accompanied the patient.
- **B2Food**: Out-of-pocket payments for additional food bought in relation to travelling the health care visit, and during visit or hospitalization, for both patient and any household member.
- **B3- Other, including accommodation:** Includes out-of-pocket payments related to renting a room/bed during health care visits, and any other non-medical payments related to health care visit, for both patient and any household member.
- **B4- Un-itemized Non-medical out of pocket payments: I**f the patient cannot distinguish between the costs for categories B1-B3, but knows the total, then leave B1-B3 blank and fill B4.
- **On this visit did you use health insurance?** Circle Yes, if the patient has health insurance and submitted the health insurance information to the facility at the beginning of the visit. Circle No, if the patient does not have health insurance or has health insurance and did not submit the information to the facility. Also circle No if the patient was at a medical facility where their insurance was not valid
- **C- Health insurance reimbursement:** Amount reimbursed to patient through medical insurance (private or social security) so far, does not include expected future reimbursement. This is not the amount the health insurance pays on the patient's behalf- it is the money that the health insurance company returns to the patient

3a. About how much money and time did you spend for each of these hospitalizations/emergency care/unplanned outpatient visits? (For patient, guardian and the person accompanying the Patient)

					Medical out-of-pocket payments, (Total per visit) (A)								Non-medical out-of-pocket payments, (Total per visit) (B)				Health insurance contribution	
Hospitalization or emergency care	Type of hospital (see Q1a)	# of days hospitalized (for hospitalizations only)	Travel time and time spent for visit (Hours)	Daily room charges (for hospitalizations only) A1	Consultation fee (total for stay) A2	Radiography and other imaging A3	Lab tests A4	Other procedure A5	Medicines (do not include TB medicines) A6	TB medicines (do not include free meds) A7	Other medicines, incl nutritional supple-ments A8	Un-itemized medical payments A9	Travel B1	Food during hospital stay B2	Other, including accommo-dation B3	Un-itemized Non-medical out-of-pocket payments B4	On this visit did you use health insurance?	Health insurance reimbursement to patient (C)
1st																	Y/N	
2nd																	Y/N	
3rd																	Y/N	
4th																	Y/N	
5th																	Y/N	
6th																	Y/N	
7th																	Y/N	
8th																	Y/N	
9th																	Y/N	
10th																	Y/N	
11st																	Y/N	
12nd																	Y/N	

	Part III: Costs for DOT and food costs during ambulatory care<br>*(DOT (Directly observed treatment) visit is for the supervision of daily intake of medicines, i.e. what is done every day. These questions are not referring to less frequent trips to pick up drugs (e.g., weekly), which are explored from Q5 onwards.)*		
4.	**On a daily basis, do you currently take your medicines yourself without supervision or support (self-administered) or do you have a treatment supervisor or supporter (DOT)?**	1. Self-administered<br>2. DOT via DTU<br>3. DOT via DTU/commune health station<br>4. DOT via private provider	• *If 1. self-administered skip to Q24*<br>• *If 2. DOT via DTU or 3. DTU/commune health station or 4.* DOT via private provider*, ask Q23a- Q23g*
4a.	**-> For any form of DOT, how many times a week?**	________ Times/week	*The maximum will be 7 times a week*
4b.	**-> Who is your DOT provider/supporter?**	1. Commune health station<br>2. DTU officer<br>3. Community health workers (CTV) or TB counsellors (TVV)<br>4. Work place<br>5. Family member<br>6. Doctor or other health care staff at the private provider<br>7. Other________________	
4c.	**-> How long did the last DOT visit take, including travel time and waiting time (total turnaround time)?**	_________Minutes	
4d.	**-> What was the cost of transport (return) for the last DOT visit, including parking costs, in total for you and any accompanying household member?**	_________ VND	
4e.	**-> How much did you spend on food, drinks and accommodation for the last DOT visit (on the road, while waiting, lunch etc.), in total for you and any accompanying household member?**	_________ VND	
4f.	**-> If there were any other fees or costs, how much did you spend on other fees during your last DOT visit?**	_________ VND	*Include: plastic bags for sputum transport, cost of injections, travel for a DTU officer to the patient's home, etc..*<br>*If no other costs, then answer 0 VND*
4g.	**-> Did somebody in your household accompany you for your last DOT visit?**	1. Yes<br>2. No	

	Part IV: Costs of picking up drugs and food costs during outpatient care<br>*This section does not concern DOT visits, which should have recorded in the last section, but should filled if patient or other household member picks up drugs for either bringing to DOT provider or for self-administered treatment.*		
5.	**Do you or a household member pick up TB drugs (for self-administered treatment or to bring to your DOT supervisor/supporter)?**	1. Yes<br>2. No	*If patient is on DOT and patient or household member is **not** picking up drugs to bring to DOT provider, then the answer is no.*<br>*If yes, then answer 5a.-5h.*<br>*If no, skip to Q6 on page 6*
5a.	**-> How often do you or a household member pick up TB drugs in the current treatment phase?**	1. Every day<br>2. Every week<br>3. Every 2 weeks<br>4. Every month<br>5. Other_____________	
5b.	**->Where do you or your household member pick up your TB drugs?**	1. Commune health station<br>2. District TB unit<br>3. Other:_________	*If the patient has visited different places, tick the most recent one.*
5c.	**-> What accommodation cost did you and any accompanying household member have when you last picked up drugs?**	_________ VND	
5d.	**-> How long did the last visit to pick up drugs take, including travel time and waiting time (total turnaround time)?**	_________Minutes	
5e.	**-> What was the cost of transport (return) last time you picked up drugs, including parking costs, in total for you and any accompanying household member?**	_________ VND	
5f.	**-> How much did you spend on food and drinks last time you picked up drugs (on the road, while waiting, lunch etc.), in total for you and any accompanying household member?**	_________ VND	
5g.	**-> If there were any other fees or costs, how much did you spend on these other fees the last time you picked up drugs, in total, for you and any accompanying household member?**	_________ VND	*If no other costs, then answer 0 VND*
5h.	**-> Did somebody in your household accompany you for your last visit to pick up drugs or did they pick up drugs for you?**	1. Yes<br>2. No	

	Part V: Cost during outpatient visits for medical follow-up (see the doctor or nurse, have tests)		
6.	**How many TB-related medical follow-up visits have you had since the last interview (to see the doctor or nurse, have follow- up tests, etc.)?**	_________ Times	*This concerns medical follow-up visits or follow-up tests that are scheduled by the doctor as part of the TB treatment. It does not include DOT visits or visits to pick up drugs or unplanned outpatient visits.*
7.	**How long did the last follow-up medical outpatient visit take, including travel time and waiting time (total turnaround time)?**	_________ Minutes	
8.	**What was the cost of transport (return) at the last follow-up medical outpatient visit, including parking, in total for you and any accompanying household member?**	_________ VND	*Cost related to the latest visit. If the interview takes place at the end of such a visit use the costs for the present visit for Q8-18.*
9.	**How much did you spend on food and drinks at the last follow-up medical outpatient visit (on the road, while waiting, lunch etc.), in total for you and any accompanying household member?**	_________ VND	
10.	**What accommodation cost did you have for the last follow-up medical outpatient visit, in total, for you and any accompanying household member?**	_________ VND	
11.	**What fees did you pay during your last follow-up medical outpatient visit for registration/consultation?**	_________ VND	
12.	**What fees did you pay during your last follow-up medical outpatient visit for radiography and other imaging?**	_________ VND	
13.	**What fees did you pay during your last follow-up medical outpatient visit for tests, TB tests and others?**	_________ VND	
14.	**What fees did you pay during your last follow-up medical outpatient visit for other procedures?**	_________ VND	
15.	**What fees did you pay at your last follow-up medical outpatient visit for TB medicines, including prescriptions for medicines bought outside the facility?**	_________ VND	
16.	**What fees did you pay during your last follow-up medical outpatient visit for other medicines, including nutritional supplements?**	_________ VND	
17.	**What other fees not listed in the previous questions did you pay during your last follow-up medical outpatient visit?**	_________ VND	*Examples include: masks, health staff fee (unofficial)*
18.	**Did somebody in your household accompany you for your last medical follow-up visit?**	1. Yes<br>2. No	

	Part VI: Costs for nutritional/food supplements		
19.	**Do you buy any nutritional supplements outside your regular diet because of the TB illness, for example vitamins, meat, energy drinks, or fruits as recommended by health care staff?**	1. Yes<br>2. No	*If yes, ask Q19a*<br>*If no, skip to Q20*
19a.	**-> How much did you spend on nutritional supplements (vitamins, meat, energy drinks, or fruits) in the past week approximately?**	________ VND	Record here the actual costs for supplements in the past week.
	Part VII: Time loss for guardians<br>• ***Not to be filled if the patient is under 15 years – for children, all questions concerning costs, time spent, income, and income loss concern cost for the guardian.***<br>• ***Note: out-of-pocket costs of transport, food, accommodation for guardian should be included in questions on Part VI.***		
20.	Earlier in the survey, you indicated that others have accompanied you as you sought care for TB and we collected the out-of-pocket costs for the person who accompanied you**. Can you identify the last time someone accompanied you as you sought healthcare and the type of visit that you were going for?**	0. No one has accompanied me<br>1. Hospitalization/emergency care/unplanned outpatient visit<br>2. DOT visit<br>3. Drug pick-up<br>4. Medical follow-up visit	*If yes, to Q4g, Q5h or 18 then responses to Q20 should be 1-4.*<br>*If Q20 is 0. No one accompanied me, then skip to Q21*
20a.	**->Who accompanied you to that visit?**	1. Grandfather<br>2. Grandmother<br>3. Father<br>4. Mother<br>5. Uncle<br>6. Aunt<br>7. Husband<br>8. Wife<br>9. Brother<br>10. Sister<br>11. Cousin (male)<br>12. Cousin (female)<br>13. Child (son)<br>14. Child (daughter)<br>15. Friend<br>16. Other household member_____	
20b.	**->During that last visit, did the person who accompanied you, take time off work?**	1. Yes<br>2. No	
20c.	**->Did that person lose any income as they accompanied you for the visit?**	1. Yes<br>2. No	*If Q20c is 1. Yes, then ask 20d.*<br>*If Q20c is 2. No, then skip to Q21*
20d.	**->How much income would you estimate that the person who accompanied you lost during your last visit?**	________ VND	

	Part VIII: Income (reported) & Income changes & social consequences		
21.	**What is your main occupation now?**	1. School student<br>2. Technician<br>3. Service<br>4. Factory worker<br>5. Farmer<br>6. Government employee<br>7. Teacher<br>8. Retiree<br>9. Homemaker<br>10. Unemployed<br>11. Manual labor<br>12. Microenterprise owner<br>13. Other (specify): _____	*If patient is under 15 years, this question is for the guardian.*
22.	**What is your primary employment now?**	0. Unemployed<br>1. Formal paid work<br>2. Informal paid work<br>3. Retired<br>4. Student<br>**5.** Housework<br>6. Other (specify): ______	*If patient is under 15 years, this question is for the guardian.*<br><br>*"3. Retired" is defined as when a person is no longer working because of their age. It does not only refer to government employees who receive a pension.*
23.	**Are you currently working?**	1. Yes<br>2. No	*If Q23 is 1. Yes, then ask Q23a-23c.*<br>*If Q23 is 2. No, then ask Q23d -23f.*
23a.	**-> If yes, how many hours per week are you working now?**	_________ hours/week	*Ask if Q23 is 1.Yes*
23b.	**-> If yes, what is your <u>current</u> monthly take-home income (net)?**	__________ VND	*Ask if Q23 is 1.Yes*
23c.	**-> If yes, how many working days have you lost because of TB since our last interview?**	_________ Days	*Ask if Q23 is 1. Yes*<br>*Working days: e.g., if a patient was not able to work for 5 half days and lost income for these, the number of days lost is 0.5*5=2.5.*
23d.	**-> If no, when did you stop working?**	_____/_____/_____	*Ask if Q23 is 2. No*<br>*Day/month/year*
23e.	**-> If no, why did you stop working?**	1.Illness related to TB<br>2. Not related to TB	*Ask if Q23 is 2. No*
23f.	**-> If no, how many working days did you lose before you stopped working entirely?**	_________ Days	*Ask if Q23 is 2. No*<br>*Working days: e.g., if a patient was not able to work for 5 half days and lost income for these, the number of days lost is 0.5*5=2.5.*<br>*Report for all days before job loss.*

24.	We are interested in understanding the current income of your household. We previously listed all of the people in your household during the first interview. **Can you estimate how much income each person in your household currently earns (take-home income or net) per month from labour related activities?**<br><br>*Enter:* Estimated current net income from labour related activities of the household per month:	_________ VND	*The interviewer should go through the list of people created in the first interview, which has the estimated income for each person from Q54 in the first interview The interviewer should ask if each person on the list is currently working. The interviewer should write down the estimated income from labour related activities for each person. Once the interviewer has obtained an estimated current income for each household member, the interviewer should add the estimated income for each person in the household and enter the total here.*
25	**Can you estimate how much income each person in your household currently earns (take-home/net income) per month from non-labour related activities?**	________ VND	*Include income from owning rentals, interest from investments, etc… If no other income, mark 0 VND.*
26.	**Did you or your household receive any social welfare payment after you were diagnosed with TB?**	0. No<br>1. Paid sick leave<br>2. Disability grant<br>3. Cash transfer for poor families<br>4. Cash transfer for other preferential policies<br>5. Other cash transfer	*Multi-select. If answer 0. No, skip to Q60*<br>*Answer 2,3,4 refers to the frequent cash transfers provided by governmental agencies/through governmental policies. Answer 5 refers to the cash transfers provided by other types of organizations or individuals, including IRD/FIT/CHI social support program, charity programs, cash from relatives or friends who are not members of the household.*
26a.	**-> How much did you receive for paid sick leave in the last month?**	________ VND	*Ask if Q26=1, Paid Sick Leave*
26b.	**-> How much did you receive for the disability grant in the last month?**	________ VND	*Ask if Q26=2. Disability Grant*
26c.	**-> Did study officers from the social support program assist you in obtaining a disability grant?**	1. Yes<br>2. No	*Ask if Q26=2. Disability Grant*<br>*Only applicable if support was provided by IRD/FIT/CHI*
26d.	**-> If yes, how much support did the program officers help you receive from the disability grant in the last month?**	_________ VND	*Ask if Q26c=1. Yes*<br>*Only applicable if support was provided by IRD/FIT/CHI*
26e.	**-> How much did you receive for cash transfers for poor families in the last month?**	________ VND	*Ask if Q26=3. Cash transfer for poor families*
26f.	**-> Did the study officers from the social support program assist you in obtaining any cash transfers for poor families?**	1. Yes<br>2. No	*Ask if Q26=3. Cash transfer for poor families*<br>*Only applicable if support was provided by IRD/FIT/CHI*
26g.	**-> If yes, how much support did the program officers help you receive from the cash transfers for poor families in the last month?**	________ VND	*Ask if Q26f=1. Yes;*<br>*Only applicable if support was provided by IRD/FIT/CHI*
26h.	**-> How much did you receive for other cash transfers in the last month?**	________ VND	*Ask if Q26=4. Include payments to elderly people and any other cash transfers from other preferential policies*
26i.	**-> Did the study officers from the social support program assist you in obtaining any other cash transfers?**	1. Yes<br>2. No	*Ask if Q26=4 Other cash transfers. Only applicable if support was provided by IRD VN/FIT/CHI*
26j.	**-> If yes, how much support did the program officers help you receive from other cash transfers in the last month?**	________ VND	*Ask if Q26i=1. Yes*<br>*Only applicable if support was provided by IRD/FIT/CHI*

26k.	**How much did you receive for other routine cash transfers in the last month?**	________ VND	*Ask if Q26=5*
26l.	**How much did you receive in total from one-time and infrequent cash transfers since the last interview?**	________ VND	*Ask if Q26=5. All one-time and infrequent cash transfers should be recorded here. Do not include any payments which have been recorded in 26a-k.*
27.	**Do you currently receive vouchers or goods in kind to cope with TB illness?**	1. Yes<br>2. No	*If no, skip to Q28.*
27a.	**-> From whom do you receive the voucher/ goods**	1. Government<br>2. NGO<br>3. Employer<br>4. Private donation<br>5. Other: _________	*Ask if Q27 is 1. Yes*<br>*Multi-select*<br>*Vouchers and goods provided by IRD/FIT/CHI will be counted as NGO.*
27b.	**-> How much do you receive per month in travel vouchers?**	_________ VND	*Ask if Q27 is 1.Yes*
27c.	**-> Did the program officers from the social support program assist you in obtaining these travel vouchers?**	1. Yes<br>2. No	*Ask if Q27b >0. Only applicable if support was provided by IRD VN/FIT/CHI*
27d.	**-> If yes, how much support did the program officers help you receive from travel vouchers in the last month?**	_________ VND	*Ask if Q27c. is 1. Yes.*<br>*Only applicable if support was provided by IRD VN/FIT/CHI*
27e.	**-> How much do you receive per month in food support?**	_________ VND	*Ask if Q27 is 1.Yes*
27f.	**-> Did the program officers from the social support pilot assist you in obtaining this food support?**	1. Yes<br>2. No	*Ask if Q27e >0. Only applicable if support was provided by IRD VN/FIT/CHI*
27g.	**-> If yes, how much support did the program officers help you receive the food support in the last month?**	_________ VND	*Ask if Q27f. is 1.Yes*
27h.	**-> How much do you receive per month in other enablers?**	_________ VND	*Ask if Q27 is 1.Yes*
27i.	**-> Did the program officers from the social support program assist you in obtaining these other enablers?**	1. Yes<br>2. No	*Ask if Q27h >0. Only applicable if support was provided by IRD VN/FIT/CHI*
27j.	**-> If yes, how much did the program officers help you receive the other enablers in the last month?**	_________ VND	*Ask if Q27i. is 1. Yes*<br>*Only applicable if support was provided by IRD VN/FIT/CHI*
27k.	**How much did you receive from one-time and infrequent vouchers and in-kind support since the last interview?**	________ VND	*Ask if Q27 is 1. Yes.*<br>*All one-time and infrequent vouchers and in-kind support should be recorded here, including gifts from relatives and friends who are not members of the household, charity, association. Do not include any in-kind transfers which have been recorded in 27b-j.*

28.	**How many people are in your household?**	_______ people	*The interviewer should review the list the members of the household from the first interview and assess whether the household size has changed since the last interview.*
28a.	**-> Number of adults**	_________ Adults	
28b.	**-> Number of children**	_________ Children	
29.	**Besides yourself, does anyone else of your household receive treatment for TB?**	1. Yes<br>2. No	Active TB treatment only. Do not include LTBI treatment.
29a.	**->How many people in your household receive treatment for DS-TB?**	_________ people	Ask if Q29 is 1.Yes
29b	**->How many people in your household receive treatment for DR-TB?**	_________ people	Ask if Q29 is 1.Yes
29c	**-->How many people in your household receive treatment for LTBI?**	_________ people	Ask if Q29 is 1.Yes
30.	**Has the TB illness affected your social or private life in any way?**	1. No<br>2. Food insecurity<br>3. Divorce or Separated from spouse/partner<br>4. Loss of Job<br>5. Interrupted schooling<br>6. Social exclusion<br>7. Isolation from family<br>8. Experienced stigma<br>9. Self-limiting contact/ communication with others<br>10. Other_________	More than one category allowed.
	Part IX: Coping		
31.	**Did you borrow or receive any money to cover costs incurred since the last interview?**	1. Yes<br>2. No	*Pawning items should be included here since it is a loan secured by an asset.*<br>*If Q31. Is 1. Yes, ask Q31a and Q31b*<br>*If Q31 is 2. No, then skip to Q32.*
31a.	**-> If yes, how much did you borrow (in total) or receive since the last interview?**	_________ VND	*Ask If Q31 is 1.Yes*
31b.	**-> From whom did you borrow?**	1. Family<br>2. Neighbors/friends<br>3. Private bank<br>4. Cooperative<br>5. Employer<br>6. "Unofficial lender" (Black market)<br>7. Pawnshop<br>8. Other: _________	*Ask If Q31 is 1. Yes. Multiple responses allowed.*<br>*If the patient took out a loan at a state-owned commercial bank, including*<br>- *Vietnam Bank for Agriculture and Rural Development - Agribank,*<br>- *Global Petro Sole - GP Bank,*<br>- *Ocean Bank,*<br>- *Construction Bank,*<br>*choose 8. Other and specify the bank*

32.	**-> If you took out any loans since TB treatment began, have you started paying back the loan(s)?**	1. Yes<br>2. No	*Some loans may have been recorded in the first interview. This question should capture repayment of both the loans borrowed in Q64 of the 1st interview and Q31 in the 2nd or 3rd interviews*
32a.	**-> How much of the principle have you paid back since the last interview?**	________ VND	*Ask If Q32 is 1.Yes*
32b.	**-> How much of the interest have you paid back since the last interview?**	________ VND	*Ask If Q32 is 1.Yes*
33.	**Have you sold any of your property to finance the costs incurred during TB treatment?**	1. Yes<br>2. No	*If 1. Yes, then ask Q 33a-33e.*<br>*If 2.No, then skip to Q34.*
33a.	**-> If yes, what did you sell?**	1. Land<br>2. Livestock<br>3. Transport/vehicle<br>4. Household item<br>5. Farm product<br>6. Gold/jewelry<br>7. Other :________	*Ask if Q33 is 1. Yes*<br>*Multiple responses allowed. Circle all that are mentioned*
33b.	**-> How much money did you receive from the sale of all items of your property (in total)?**	________ VND	*Ask if Q33 is 1.Yes*
33c.	**The assets that you sold, were they previously supporting the family income (or expenditure)?**	1. Yes<br>2. No	*Ask if Q33 is 1.Yes*
33d.	**-> If yes indicate monthly income previously generated by the assets**	________ VND	*Ask if Q33c is 1.Yes*
33e.	**What is the estimated market value of all the property you sold?**	________ VND	*Ask if Q33 is 1.Yes*<br>*Market value refers to the value that could have been realized if the seller was not under pressure to sell quickly to raise funds.*

34.	**Thank you for your cooperation! Is there anything you would like to ask or say?**		
**35.**	**Comments by Interviewer:**		
**Date** *(dd/mm/yyyy)*:	____/____/____	**Signature interviewer:**	____
