# Supplementary material for: Determinants of catastrophic costs among households affected by multi-drug resistant tuberculosis in Ho Chi Minh City, Viet Nam: a prospective cohort study
Source: BMC Public Health. 2023 Dec 3;23:2372. doi: 10.1186/s12889-023-17078-5 (PMC10693707; doi:10.1186/s12889-023-17078-5)
Supplement: Supplementary file 5 — Additional file 5. Description of alternative estimation approaches of catastrophic costs. [file 12889_2023_17078_MOESM5_ESM.pdf]

Description of alternative estimation approaches of catastrophic costs

Primary Analysis

The output approach estimates indirect costs based on the self-reported income before the start of TB treatment and at different interview timepoints during the treatment. The difference in income of each timepoint was summed, extrapolated to the individual's entire treatment duration, and noted as total income loss during the entire treatment. If the difference was zero or above, income loss was noted as zero.

Total costs in the numerator included expenses that arose during ambulatory-care, hospitalisations, and follow-up visits, as well as visits for directly observed therapy (DOT) and drug pick-up. Missing values for single cost components were imputed with the median if less than 10% were missing. Reimbursements from social health insurance was subtracted from direct non-medical costs. All cost components in the numerator were summed up, and extrapolated to the individual's entire treatment duration.

In the denominator, annualised self-reported household income, and amounts received from any social protection mechanism such as cash transfers or vouchers were summed.

Finally, if the percentage of total TB related costs exceeds 20% of the affected household's annual income, the individual was ladled positive for the occurrence of catastrophic costs.

Alternative I:

As in the primary analysis, the output approach was used for the estimation of indirect costs. Yet, here we excluded the amounts received from any social protection mechanism such as cash transfers or vouchers from the denominator.

Alternative II:

As in the primary analysis, the output approach was used for the estimation of indirect costs. Instead of using self-reported household income in the denominator, total household income is modelled by a proxy measure of self-reported household assets and dwelling characteristics. A linear regression between household income before the treatment and all asset variables was used with a stepwise forward selection approach, removing terms with $p \geq 0.2$.

Alternative III:

The human capital approach is an alternative approach to estimate indirect costs. The estimation is based on time loss by multiplying total hours lost due to care-seeking during the entire TB

episode by hourly wage. If hours worked was not reported, a 40-hour working week was assumed.

Alternative IV:

To investigate the impact of direct cost on the occurrence of catastrophic costs, we excluded the indirect cost component from the nominator.
